# Macrophage hypoxia signaling regulates cardiac fibrosis via Oncostatin M

**DOI:** 10.1038/s41467-019-10859-w

**Published:** 2019-06-27

**Authors:** Hajime Abe, Norihiko Takeda, Takayuki Isagawa, Hiroaki Semba, Satoshi Nishimura, Masaki Suimye Morioka, Yu Nakagama, Tatsuyuki Sato, Katsura Soma, Katsuhiro Koyama, Masaki Wake, Manami Katoh, Masataka Asagiri, Michael L. Neugent, Jung-whan Kim, Christian Stockmann, Tomo Yonezawa, Ryo Inuzuka, Yasushi Hirota, Koji Maemura, Takeshi Yamashita, Kinya Otsu, Ichiro Manabe, Ryozo Nagai, Issei Komuro

**Affiliations:** 1 Department of Cardiovascular Medicine, Graduate School of Medicine, The University of Tokyo, 7-3-1 Hongo, Bunkyo-ku, Tokyo, 113-8655 Japan; 2 The School of Cardiovascular Medicine and Sciences, King’s College London British Heart Foundation Centre of Excellence, London, SE5 9NU UK; 3 PRESTO, JST, 4-1-8 Honcho Kawaguchi, Saitama, 332-0012 Japan; 4 Graduate School of Biomedical Science, Nagasaki University, 1-7-1sakamoto, Nagasaki, 852-8501 Japan; 5 Department of Cardiovascular Medicine, The Cardiovascular Institute, 3-2-19 Nishiazabu, Minato-ku, Tokyo, 106-00031 Japan; 6 Center for Molecular Medicine, Jichi Medical University, 3311-1 Yakushiji, Shimotsuke-shi, Tochigi, 329-0498 Japan; 7 Depertment of Bioinformatics, Medical Research Institute, Tokyo Medical and Dental University, 1-5-45 Yushima, Bunkyoku, Tokyo, 113–8510 Japan; 8 Department of Pediatrics, Graduate School of Medicine, The University of Tokyo, 7-3-1 Hongo, Bunkyo-ku, Tokyo, 113-8655 Japan; 9 Department of Pathobiology, Graduate School of Pharmaceutical Sciences, Nagoya City University, 3-1 Tanabe-dori, Mizuho-ku, Nagoya, 467-8603 Japan; 10 Department of Biological Sciences, The University of Texas at Dallas, 800W. Campbell Road FO 3.704G, Richardson, TX 75080 USA; 11 Institute of Anatomy, University of Zurich, Zurich, CH-8057 Switzerland; 12 Cancer Research Center Zurich, Winterthurerstrasse 190, CH-8057 Zurich, Switzerland; 13 Center for Therapeutic Innovation, Gene Research Center, Center for Frontier Life Sciences, Nagasaki University, Graduate School of Biomedical Sciences, 1-12-14 Sakamoto, Nagasaki, 852-8523 Japan; 14 Department of Obstetrics and Gynecology, Graduate School of Medicine, The University of Tokyo, 7-3-1 Hongo, Bunkyo-ku, Tokyo, 113-8655 Japan; 15 Department of Disease Biology and Molecular Medicine, Graduate School of Medicine, Chiba University, 1-8-1 Inohana, Chuo-ku, Chiba-shi, Chiba, 260-8670 Japan; 16 Jichi Medical University, 3311-1 Yakushiji, Shimotsuke-shi, Tochigi-ken, Tochigi, 329-0498 Japan

**Keywords:** Macrophages, Heart failure, Hypoxia

## Abstract

The fibrogenic response in tissue-resident fibroblasts is determined by the balance between activation and repression signals from the tissue microenvironment. While the molecular pathways by which transforming growth factor-1 (TGF-β1) activates pro-fibrogenic mechanisms have been extensively studied and are recognized critical during fibrosis development, the factors regulating TGF-β1 signaling are poorly understood. Here we show that macrophage hypoxia signaling suppresses excessive fibrosis in a heart via oncostatin-m (OSM) secretion. During cardiac remodeling, Ly6C^hi^ monocytes/macrophages accumulate in hypoxic areas through a hypoxia-inducible factor (HIF)-1α dependent manner and suppresses cardiac fibroblast activation. As an underlying molecular mechanism, we identify OSM, part of the interleukin 6 cytokine family, as a HIF-1α target gene, which directly inhibits the TGF-β1 mediated activation of cardiac fibroblasts through extracellular signal-regulated kinase 1/2-dependent phosphorylation of the SMAD linker region. These results demonstrate that macrophage hypoxia signaling regulates fibroblast activation through OSM secretion in vivo.

## Abstract (web summary)

Fibrosis is a hallmark of several cardiac pathologies and its underlying mechanisms are still poorly defined. Here the authors show that macrophage hypoxia signaling following transverse aortic constriction in mice suppresses the activation of cardiac fibroblasts by secreting oncostatin M.

## Introduction

Cardiac remodeling occurs after myocardial infarction or pressure overload. During the remodeling processes, activated fibroblasts (myofibroblasts) cause cardiac fibrosis development in the infarcted areas of the myocardium in order to support the structure of the heart^1–3^. Cardiac fibrosis also occurs in pressure overload-induced cardiac remodeling, leading to the development of heart failure with preserved ejection fraction (HFpEF), a form of congestive heart failure in which the fraction of blood ejected from the left ventricle is within normal thresholds (>50%)^4^. In both myocardial infarction and pressure overload-induced cardiac fibrosis, the extent of interstitial fibrosis correlates with mortality and major cardiovascular adverse event rates in heart failure patients^5–8^. Therefore, elucidation of the molecular processes by which fibroblasts are activated or deactivated is critically important for the development of therapeutic approaches in the management of cardiac fibrosis.

During fibrogenesis, several subsets of monocytes/macrophages (MΦ) are recruited to the heart including both pro-inflammatory (Ly6C^hi^) and anti-inflammatory (Ly6C^lo^) MΦ^9^ and play a critical role in tissue remodeling. The extent of inflammation significantly correlates to the severity of heart failure^10–12^. Notably, local oxygen concentration within inflamed areas tends to be decreased, leading to tissue hypoxia^13,14^. The transcription factor, hypoxia-inducible factor (HIF)-1α and HIF-2α are stabilized under hypoxic conditions. In its stable form, HIF-αs induce target gene expression through their binding to the hypoxia response elements (HREs) of target genes^15–23^. We previously showed that HIF-1α and HIF-2α are highly expressed in pro-inflammatory and anti-inflammatory MΦ, respectively^24^. During cell migration, cytosolic ATP is rapidly consumed at the filopodia or lamellipodia. Recently, we revealed that HIF-1α mediated glycolytic reprogramming plays a key role for MΦ to mobilize towards the hypoxic tissue^25^. Pyruvate kinase, muscle type, a glycolytic enzyme capable of producing ATP, localizes in the filopodia and lamellipodia, which may account for the beneficial roles of glycolytic metabolism in MΦ migration.

The roles of MΦ, however, in tissue fibrosis have not been fully elucidated^9,26^. The current study examine the roles of MΦ in cardiac fibrosis using murine model of cardiac remodeling. The results demonstrate that Ly6C^hi^ MΦ accumulate in hypoxic areas and suppress excessive fibrosis in cardiac tissue by secreting a cytokine Oncostatin-M (OSM) in vivo.

## Results

### Ly6C^hi/lo^ MΦ accumulate to the heart after TAC procedure

**Fig. 1:**
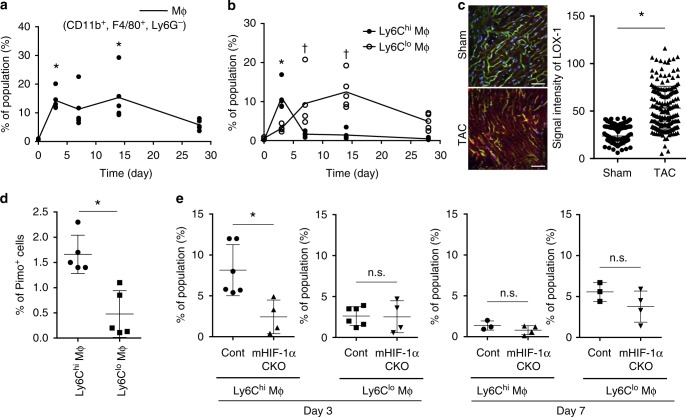
Cardiac infiltration of MΦ after TAC operation. **a** Data represent the cardiac infiltration of MΦ (CD11b^+^, F4/80^+^, Ly6G^−^ mononuclear cells) after TAC operation. **b** Data represent the cardiac infiltration of Ly6C^hi^ MΦ and Ly6C^lo^ MΦ after TAC operation. *Ly6C^hi^ MΦ. ^†^Ly6C^lo^ MΦ. The Kruskal–Wallis test was used for the statistical analysis. (Figure **a**, **b**; *n* = 5 at day 0, 3, 7, 14, and 28). **c** Hypoxic area was visualized in TAC operated mice (3 days after TAC) using LOX-1 (red). Vasculatures and nucleus were labeled with isolectin (green) and Hoechst 33342 (blue) (left). Scale bar = 50 μm. LOX-1 signal intensity was calculated in sham or TAC operated mouse (right). (ROI number 180, sham and TAC operated mouse, respectively). **d** MΦ after TAC operation were stained with Pimonidazole (Pimo). The population of Pimo positive cells in Ly6C^hi^ MΦ (day 3, *n* = 5) and Ly6C^lo^ MΦ (day 7, *n* = 5) are shown. **e** Cardiac infiltration of Ly6C^hi^ MΦ and Ly6C^lo^ MΦ in myeloid-specific HIF-1α conditional knockout mice (*mHIF-1α CKO: HIF-1α*^*flox/flox*^*; LysM-cre*^*+/−*^) were analyzed 3 or 7 days after TAC operation. Cre negative littermates were used as a control (cont). Day 3, cont (*n* = 6) and mHIF-1α CKO (*n* = 4). Day 7, cont (*n* = 3) and mHIF-1α CKO (*n* = 4). The Mann–Whitney U test was used to compare differences (**c**, **d**, **e**). Error bar represents the standard deviation. n.s. not statistically significant. * or ^†^, *p* < 0.05

TAC is a commonly used experimental model for inducing cardiac fibrosis. To identify the population of cardiac inflammatory cells which accumulates in the heart tissue, we performed fluorescence-activated cell sorting (FACS) analysis of cardiac tissues after administering the TAC operation. In this study, we defined cardiac MΦ as CD11b^+^, F4/80^+^, Ly6G^−^mononuclear cells^27^. Cardiac MΦ acutely, but transiently accumulate in the heart after TAC (Fig. 1a). Ly6C is known as a surface marker for defining cardiac MΦ subpopulations^28^. We next isolated two MΦ subsets including Ly6C^hi^ MΦ and Ly6C^lo^ MΦ (Supplementary Fig. 1)^9,29^. Cardiac Ly6C^hi^ MΦ highly express *Interleukin 1 beta* (*Il1b*) and *C-C chemokine receptor type 2* (*Ccr2*), whereas Ly6C^lo^ MΦ express *Mannose receptor, C type 1* (*Mrc1*) and *Arginase 1* (*Arg1*) (Supplementary Fig. 2). Most of the cells recruited at day 3 were Ly6C^hi^ MΦ, and day 7 or 14 were Ly6C^lo^ MΦ (Fig. 1b).

### Ly6C^hi^ MΦ accumulate in hypoxic regions

Chemokine (C-C motif) ligand 2 (Ccl2) and chemokine (C-X3-C motif) ligand 1 are chemokines, which have a potential to recruit Ly6C^hi^ and Ly6C^lo^ MΦ, respectively^9^. While the recruited MΦ presumably modulates cardiac fibrosis, its migration process is significantly influenced by tissue oxygenation^14,25^. To test whether hypoxic areas develop during cardiac remodeling, we performed in vivo two photon microscopic analyses using a phosphorescent hypoxia probe, LOX-1. Striking augmentation of LOX-1 signal was observed at 3 days after TAC operation, reflecting tissue hypoxia (Fig. 1c). We used another hypoxia probe, pimonidazole (Pimo), to further examine the oxygenation status of cardiac tissues^30^. Pimo staining was visualized in the whole area of the heart at 3 days after TAC (Supplementary Fig. 3). To clarify the molecular link between mechanical stress and tissue hypoxia, we isolated primary cardiomyocytes and measured the oxygen consumption rate (OCR) using flux analyzer (XF24, Agilent Technologies, USA). OCR of the cardiomyocytes was increased at day 3 after TAC operation (Supplementary Fig. 4). These results indicate a hypothesis that elevated oxygen demand in cardiomyocytes underlies the occurrence of tissue hypoxia in pressure overloaded heart.

We next measured Pimo signals in cardiac MΦ. Ly6C^hi^ MΦ displayed a higher Pimo signal intensity than Ly6C^lo^ MΦ, suggesting that Ly6C^hi^ MΦ indeed accumulate in hypoxic areas in vivo (Fig. 1d). To test the roles of HIF-1α signaling in cardiac MΦ distribution, we generated myeloid-specific HIF-1α knockout mice (*HIF-1α*^flox/flox^; *LysM-cre*^+/−^, mHIF-1α CKO)^31,32^. The deletion efficiency of the HIF-1α gene at the mRNA level was more than 65% in both Ly6C^hi^ MΦ and Ly6C^lo^ MΦ in the heart (Supplementary Fig. 5). The baseline cardiac function was unaltered in mHIF-1α CKO mice compared with cre negative controls (Supplementary Fig. 6). We next performed the TAC procedure in mHIF-1α CKO mice. The results showed that the cardiac accumulation of Ly6C^hi^ MΦ at day 3 was strikingly decreased in mHIF-1α CKO, whereas that of Ly6C^lo^ MΦ was not affected (Fig. 1e). These results show that HIF-1α signaling plays an integral role in the recruitment of Ly6C^hi^ MΦ to the hypoxic area of the heart, but not of Ly6C^lo^ MΦ.

### Cardiac fibrosis develops in HIF-1α knockout mice

**Fig. 2:**
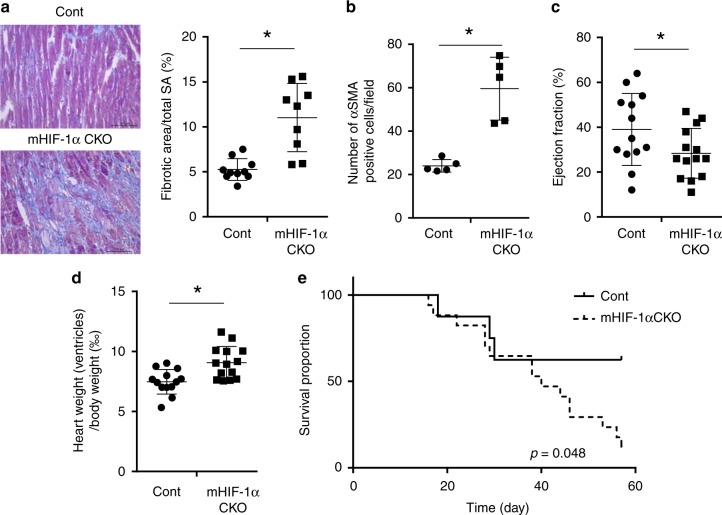
Cardiac fibrosis develops in myeloid-specific HIF-1α conditional knockout mice. **a** Masson’s trichrome staining was performed using cardiac tissue of myeloid-specific HIF-1α conditional knockout mice (*mHIF-1α CKO: HIF-1α*^*flox/flox*^*; LysM-cre*^+/−^) 14 days after TAC operation. Cre negative littermates were used as a control (cont). Fibrotic area was calculated compared to the total surface area (SA). Cont (*n* = 10) and mHIF-1α CKO (*n* = 9). Scale bar = 100 μm. **b** The average number of α-smooth muscle actin (αSMA) positive cells 14 days after TAC operation were counted within five fields. Cont (*n* = 5) and mHIF-1α CKO (*n* = 5). **c** Data represent the ejection fraction 28 days after TAC operation in cont (*n* = 13) and mHIF-1α CKO mice (*n* = 14). **d** Data represent the heart weight (ventricles) per body weight (‰) 28 days after TAC operation in cont (*n* = 13) and mHIF-1α CKO mice (*n* = 14). The Mann–Whitney U test was used to compare differences between mHIF-αCKO and cont (**a**, **b**, **c**, **d**). **e** The Kaplan–Meier survival curves for control mice (*n* = 8) and mHIF-1α CKO mice (*n* = 17). The log rank test was used for the statistical analysis. Error bar represents the standard deviation. *p < 0.05

We next measured the fibrotic area and found that it was significantly larger in mHIF-1α CKO mice compared with the controls (Fig. 2a). Whereas the number of CD31-positive cells remained unchanged, the number of alpha-smooth muscle actin (αSMA)-positive myofibroblasts was increased in mHIF-1α CKO mice (Fig. 2b, Supplementary Fig. 7). The number of TdT-mediated dUTP nick end labeling (TUNEL)-positive cells was not increased in mHIF-1α CKO mice (Supplementary Fig. 8), indicating that loss of myeloid cell-specific HIF-1α did not affect cardiomyocyte survival in vivo. While these results indicate the anti-fibrotic activity of Ly6C^hi^ MΦ, the roles of Ly6C^lo^ MΦ in cardiac fibrosis still remained unclear. Therefore, we injected clodronate liposomes intraperitoneally to further deplete Ly6C^lo^ MΦ in mHIF-1α CKO mice. Injection of clodronate liposomes did not affect the extent of cardiac fibrosis (Supplementary Fig. 9). These results suggest that Ly6C^hi^ MΦ hypoxia signaling elicits the major role in regulating cardiac fibrosis in vivo. In addition, mHIF-1α CKO mice had significantly decreased left ventricular ejection fractions along with increased heart weights (Fig. 2c, d and Supplementary Figs. 10, 11) as well as markedly decreased survival after the TAC procedure (Fig. 2e). These data indicate that MΦ hypoxia signaling has a cardio-protective role in TAC model. To further examine the roles of MΦ HIF-1α signaling in the development of replacement fibrosis, we performed myocardial infarction model using mHIF-1α CKO mice. Fibrotic area in myocardial infarction model was significantly larger in mHIF-1α CKO mice compared with controls (Supplementary Fig. 12).

### MΦ derived Oncostatin M suppresses fibroblast activation

**Fig. 3:**
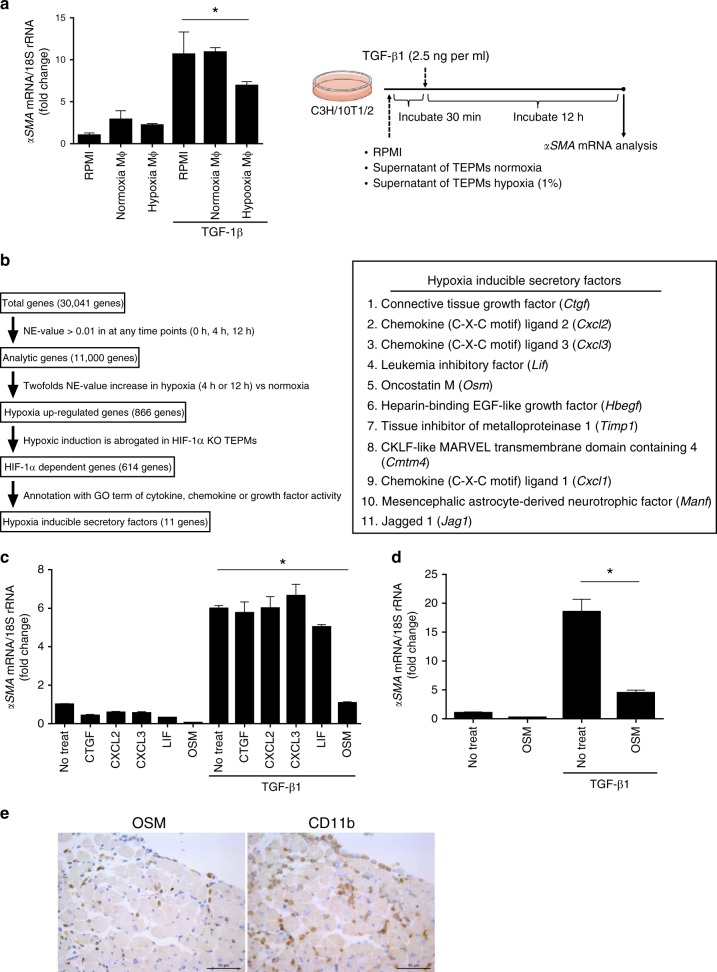
Oncostatin M from hypoxic MΦ inhibits fibroblast activation. **a** Supernatants were collected from thioglycollate-elicited peritoneal macrophages (TEPMs) under normoxic or hypoxic condition. After pretreatment with the supernatants, C3H/10T1/2 cells were stimulated with TGF-β1 (2.5 ng per ml, 12 h) and the relative expression of *αSMA* mRNA was calculated (right). The one-way ANOVA and Dunnett’s multiple comparisons test were used for the statistical analysis (F (2, 6) = 6.019). **b** We performed transcriptome analysis in isolated primary TEPMs from hematopoietic/endothelial-specific HIF-1α conditional knockout mice (*HIF-1α*^flox/flox^*; Tie2-cre*^+/−^ mice; HIF-1α KO) or cre negative littermate control. Flow chart shows the strategy to identify hypoxia-inducible secretory factors (left). Table illustrates the identified 11 hypoxia-inducible secretory factors (right). **c** The effect of each hypoxic inducible secretory factor in fibroblasts activation was examined (20 ng per ml for CTGF, CXCL2, CXCL3, LIF and 10 ng per ml for OSM). Thirty minutes after pretreatment with hypoxia-inducible secretory factors, C3H/10T1/2 cells were stimulated with TGF-β1 (2.5 ng per ml, 12 h) and the relative expression of αSMA mRNA was calculated. The one-way ANOVA and Dunnett’s multiple comparisons test were used for the statistical analysis (F (5, 12) = 68.07). **d** The effect of oncostatin M (OSM) (10 ng per ml) in primary cardiac fibroblasts activation was examined. Thirty minutes after pretreatment with OSM (10 ng per ml, 12 h), C3H/10T1/2 cells were stimulated with TGF-β1 (2.5 ng per ml, 12 h) and the relative expression of αSMA mRNA was calculated. Two-tailed *t*-test with Welch’s correction was used for the statistical analysis (t = 10.89, df = 2.208). Data show the mean and the standard deviation (error bar) of technical triplicates from a representative experiment. **e** Immunohistological staining were performed using cardiac tissues from TAC operated mice (day 3). OSM (left), CD11b (right). Scale bar = 50 μm. *p < 0.05

To elucidate the molecular mechanisms by which hypoxic MΦ regulate fibroblast activation, we established an in vitro assay system using C3H/10T1/2 cells, a mouse embryo fibroblast cell line, and thioglycollate-elicited peritoneal macrophages (TEPMs). TGF-β1 induces *αSMA* mRNA expression in C3H/10T1/2 cells, which is known as an activation marker of fibroblasts. While the culture supernatant of TEPMs kept under normoxic conditions did not alter the expression of *αSMA* mRNA, the culture supernatant of TEPMs kept under hypoxic conditions significantly suppressed *αSMA* mRNA expression (Fig. 3a). Based on this finding, we hypothesized that hypoxia stimulates the secretion of some unknown factors in TEPMs, which suppress fibroblast activation.

To identify the hypoxia-stimulated secretion factors that have anti-fibrotic potential, we isolated primary TEPMs from hematopoietic/endothelial-specific HIF-1α knockout mice (*HIF-1α*^*flox/flox*^*; Tie2-cre*^+/−^ *mice*; HIF-1α KO) or cre negative littermate controls. Subsequently, we performed transcriptome analysis and searched for any secretory factors that are highly expressed in hypoxic conditions through a HIF-1α-dependent manner (Fig. 3b, left). This unbiased approach revealed 11 secretory factors, including *Connective tissue growth factor* (*Ctgf*), *Chemokine* (*C-X-C motif*) *ligand 2* (*Cxcl2*), *Cxcl3, Leukemia inhibitory factor* (*Lif*) and *Osm* (Fig. 3b, right). To test their effects on fibroblast activation, we treated C3H/10T1/2 cells with 10 of the identified secretory factors those we could obtain on a commercial basis, and tested their effects on fibroblast activation. Through this approach, we discovered that OSM, a member of the IL6–type family of cytokines, significantly suppressed *αSMA* mRNA expression in C3H/10T1/2 cells (Fig. 3c, Supplementary Fig. 13). OSM also suppressed the activation of isolated mouse primary cardiac fibroblasts (Fig. 3d). We further tested the expression of OSM in the heart and identified that OSM is highly expressed in murine cardiac MΦ (Fig. 3e).

### OSM is induced in hypoxia through a HIF-1α dependent manner

**Fig. 4:**
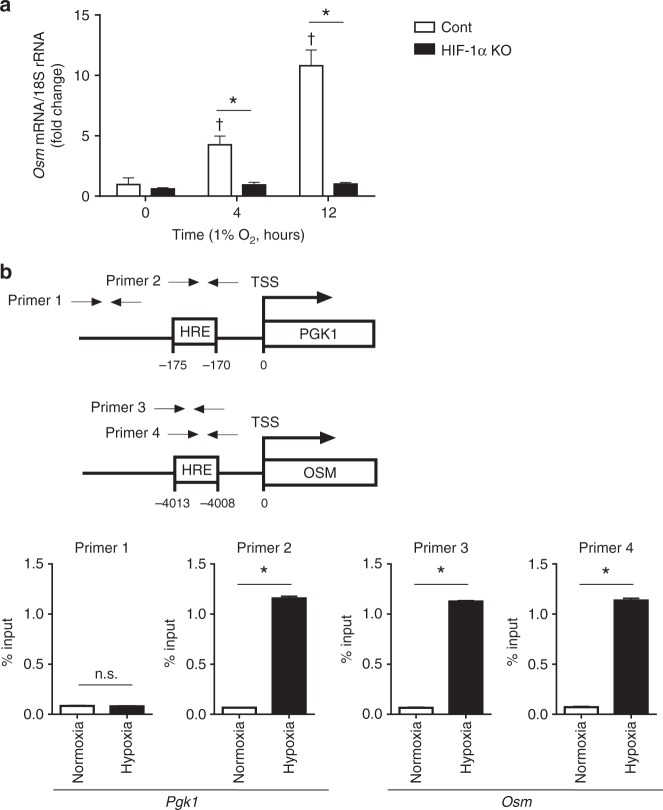
OSM gene expression is induced in hypoxia through a HIF-1α dependent manner. **a** Thioglycollate-elicited peritoneal macrophages (TEPMs) were isolated from hemtopoietic/endothelial-specific HIF-1α knockout mice (HIF-1α KO) or cre negative littermates as a control (cont). The TEPMs were exposed to hypoxia (1% O_2_), and the relative expression level of *Osm* mRNA was analyzed. The one-way ANOVA and Dunnett’s multiple comparisons test were used for the statistical analysis (F (2, 6) = 90.20). ^†^*p* < 0.05 vs 0 h. The two-way ANOVA and Sidak’s multiple comparison test was performed for the statistical analysis (F (2, 12) = 94.66). *p < 0.05 vs cont. **b** HIF-1α binding to the promoter of *Osm* gene was studied by chromatin immunoprecipitation coupled to detection by quantitative PCR. Primer set 1 and 2 were designed to detect the promoter legion (−500 and −170 bp) of *Pgk1* gene. Primer set 3 and 4 were designed to detect the hypoxia response element (HRE, −4 kb) of *Osm* gene. Data show the mean and the standard deviation (effort bar) of technical triplicates from a representative experiment. Quantitative PCR analysis were repeated at least three independent experiments. Two-tailed *t*-test with Welch’s correction was used for the statistical analysis (Primer 1 (t = 1.527, df = 3.863), Primer 2 (t = 93.69, df = 2.011), Primer 3 (t = 197.7, df = 3.202), Primer 4 (t = 75.41, df = 2.505)). n.s. not statistically significant. *p < 0.05

We cultured wild type TEPMs or bone marrow derived macrophages under 1% oxygen concentration and found that the *Osm* mRNA levels were significantly elevated under hypoxia (Fig. 4a, Supplementary Fig. 14). Hypoxia elicited elevation of *Osm* gene expression was significantly suppressed in HIF-1α KO TEPMs, indicating that the abundance of *Osm* mRNA is increased in a HIF-1α-dependent manner. To examine the molecular mechanisms by which HIF-1α induces *Osm* gene expression, we next performed chromatin immunoprecipitation (ChIP) assay with anti-HIF-1α antibody. The results showed the direct binding of HIF-1α to the HRE sequence 4 kb upstream of the *Osm* transcription start site (Fig. 4b). To evaluate the roles of *Osm* HRE sequence in its transcriptional activation, we generated a reporter construct containing the HRE sequence of *Osm*. Co-transfection of HIF-1α and aryl hydrocarbon receptor nuclear translocator significantly enhanced the luciferase activity of the reporter construct containing *Osm* HRE sequences (Supplementary Fig. 15). Together, these results demonstrate that *Osm* expression is directly induced in hypoxia through a HIF-1α-dependent manner.

### OSM inhibits SMAD signaling pathway

**Fig. 5:**
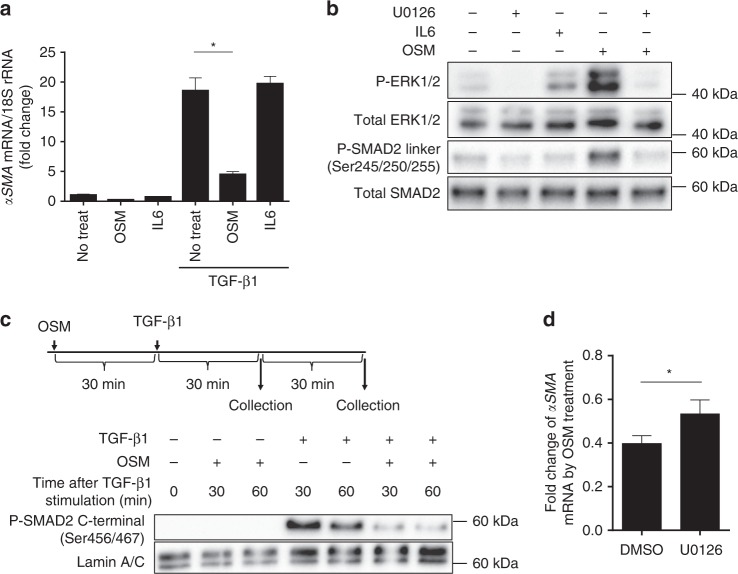
OSM suppresses SMAD signaling. **a** After pretreatment with the OSM (10 ng per ml) or IL6 (20 ng per ml), C3H/10T1/2 cells were stimulated with TGF-β1 (2.5 ng per ml, 12 h) and the relative expression level of *αSMA* mRNA was calculated. The one-way ANOVA and Dunnett’s multiple comparisons test were used for the statistical analysis (F (2, 6) = 97.35). **b** Immunoblot analysis of phospho-ERK1/2, total ERK1/2, phospho-SMAD2 linker (Ser245/250/255) and total SMAD2 protein. Thirty minutes after U0126 (20 nM) pretreatment, C3H/10T1/2 cells were stimulated with OSM (10 ng per ml) or IL6 (20 ng per ml) for 30 min. and collected for the analysis. U0126: a selective MEK1/2 inhibitor. **c** Immunoblot analysis of phospho-SMAD2 C-terminal (Ser465/467) and Lamin A/C. Thirty minutes after OSM (10 ng per ml) pretreatment, C3H/10T1/2 cells were stimulated with TGF-β1 (2.5 ng per ml, 12 h) and collected at indicated time points. **d** After pretreatment with U0126 (20 μM, 60 min) and OSM (10 ng per ml, 30 min), C3H/10T1/2 cells were stimulated with TGF-β1 (2.5 ng per ml, 12 h) and the relative expression level of *αSMA* mRNA was calculated. Two-tailed *t*-test with Welch’s correction was used for the statistical analysis (t = 3.212, df = 3.172). Data show the mean and the standard deviation (error bar) of technical triplicates from a representative experiment (**a**, **d**). *p < 0.05

We further investigated the molecular processes by which OSM decreases *αSMA* mRNA expression level in C3H/10T1/2 cells and primary cardiac fibroblasts. While OSM suppressed TGF-β1 mediated augmentation of *αSMA* mRNA expression, IL6 did not affect its abundance (Fig. 5a, Supplementary Fig. 16), suggesting that the anti-fibrotic effect is specific to OSM and not a general feature of the IL6 cytokine family. TGF-β1 signaling is mainly mediated through a canonical SMAD signaling pathway. It has been shown that extracellular signal-regulated kinase 1/2 (ERK1/2) mediated phosphorylation of the SMAD linker region suppresses its nuclear translocation^33–37^. In this study, we found that OSM activates ERK signaling in C3H/10T1/2 cells (Fig. 5b). Furthermore, OSM elicits the phosphorylation of SMAD2 linker region (Ser245/250/255), which was abolished by U0126, a selective MEK1/2 inhibitor. Notably, IL6 does not affect the phosphorylation of SMAD2 linker region. Next we extracted nuclear fractions and examined SMAD2 C-terminal (Ser465/467) phosphorylation, an activation marker of SMAD2 signaling. OSM significantly suppressed the accumulation of activated SMAD2 in the nucleus (Fig. 5c). Consistent with our hypothesis, U0126 attenuated the inhibitory effect of OSM on *αSMA* gene expression in C3H/10T1/2 cells and primary cardiac fibroblasts (Fig. 5d, Supplementary Fig. 17). These results indicate that OSM exerts its anti-fibrotic effect partly through ERK1/2-mediated phosphorylation of the SMAD2 linker region.

### OSM suppresses cardiac fibrosis in vivo

**Fig. 6:**
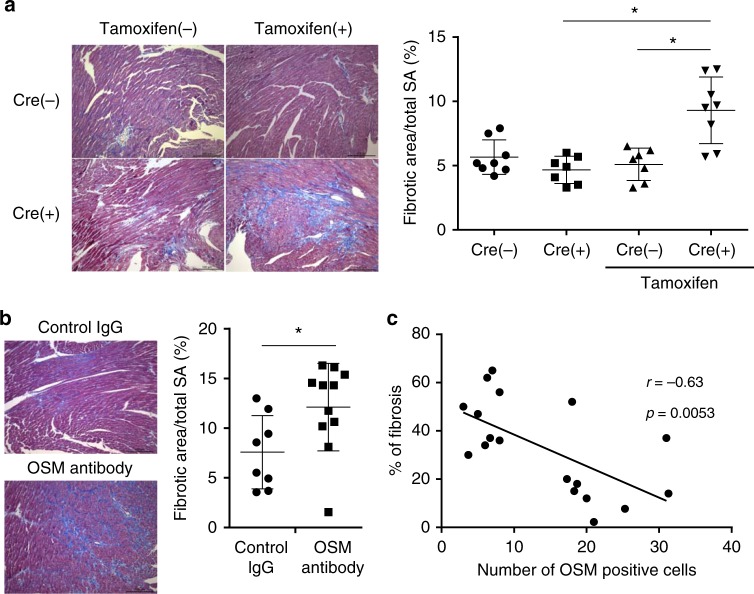
OSM suppresses cardiac fibrosis in vivo. Masson’s trichrome staining was performed using cardiac tissue of fibroblast-specific *Osmr* knockout mice (*OSMR*^*flox/flox*^*; Col1a1-creERT*, Cre(+)) 14 days after TAC operation. Cre negative littermates (Cre(−)) or tamoxifen untreated littermates were used as controls. Fibrotic area was calculated compared to the total surface area (SA) (right). The Kruskal–Wallis test was used for the statistical analysis. (Cre(−), tamoxifen(−): *n* = 8, Cre(+), tamoxifen(−): *n* = 7, Cre(−), tamoxifen(+): *n* = 7, Cre(+), tamoxifen(+): *n* = 8). Scale bar = 100 μm. **b** The effect of neutralizing antibody to OSM on cardiac fibrosis was examined. Neutralizing antibody to OSM (100 μg) or control IgG (100 μg) was injected intravenously 3 days after TAC operation. Masson’s trichrome staining was performed using cardiac tissue 14 days after TAC operation. Fibrotic area was calculated compared to the total surface area (SA). The Mann–Whitney U test was used to compare differences between the control IgG (*n* = 8) group and OSM antibody group (*n* = 11). Data show the mean and the standard deviation (error bar). Scale bar = 100 μm. **c** The correlation between the area of cardiac fibrosis and the number of OSM-positive cells was examined. Linear regression analysis was used for the correlation between percent of fibrotic area and the number of OSM-positive cells in the heart specimens from human heart failure patients (*n* = 18). *p < 0.05

It should be noted that OSM suppressed fibroblast activation, whereas IL6 did not. While OSM and IL6 share gp130 as a co-receptor for the signaling, murine OSM receptor (OSMR) is specifically activated by OSM but not IL6. To test the effect of OSM on cardiac fibroblast in vivo, we generated fibroblast-specific *Osmr* knockout mice (*OSMR*^*flox/flox*^*; Col1a1-creERT*, fOSMR CKO)^38^. The extent of baseline cardiac fibrosis or heart weight remain unchanged in fOSMR CKO mice (Supplementary Fig. 18). We next performed murine TAC operation in fOSMR CKO mice and cre-negative littermates or tamoxifen untreated mice as controls. Cardiac fibrosis was more prominent in fOSMR CKO mice than controls (Fig. 6a). To further determine the roles of OSM in vivo, we injected OSM neutralizing antibody and measured the area of cardiac fibrosis in the murine TAC model. The results showed that inhibition of OSM significantly augments the development of cardiac fibrosis (Fig. 6b). Collectively, these data suggest that OSM directly suppresses the activation of cardiac fibroblasts both in vitro and in vivo. Finally, we examined the expression of OSM in human heart specimens, which were obtained from heart failure patients. Notably, the number of OSM-positive cells in human heart specimen inversely correlated with the area of cardiac fibrosis (Fig. 6c, Supplementary Fig. 19), which is consistent with the anti-fibrotic function of OSM.

## Discussion

Cardiac fibroblasts are activated not only in myocardial infarction, but also in pressure-overload-induced cardiac hypertrophy. Despite the fact that MΦ could suppress cardiac fibrosis^9^, its regulatory signals have not been fully elucidated. Here, we used a hypoxia probe in vivo, and demonstrated that tissue hypoxia develops in pressure-overload-induced cardiac remodeling. We further discovered that hypoxic MΦ, but not normoxic MΦ, suppresses fibroblast activation through the secretion of OSM. While both OSM and IL6 transduce their signals through gp130, OSMR specifically interact with src homology 2 domain-containing transforming protein C1 (SHC1)^39^. This seems to account for the OSM mediated activation of ERK signaling, contributing to its anti-fibrotic properties. OSM was also shown to have a protective effect in ischemic myocardium^40,41^, thus Ly6C^hi^ MΦ-derived OSM exerts a cardio-protective effect on both cardiomyocytes and cardiac fibroblasts. Our results demonstrate a functional link between sterile inflammation and cardiac fibrosis.

In myocardial infarction model, Ly6C^hi^ and Ly6C^lo^ MΦ sequentially accumulate in the heart^9^. It has also been shown that CCL2 or Regenerating islet-derived 3β (Reg3β) has a strong potential to recruit Ly6C^hi^ MΦ to the damaged heart^42^. In our model, the expression of *Ccl2*, but not *Reg3β* is highly induced at day 3 after TAC (Supplementary Fig. 20). While it is uncertain whether Ly6C^hi^ and Ly6C^lo^ MΦ give rise to distinct macrophage subsets in the heart, the results of our study are consistent with the finding that Ly6C^hi^ MΦ are recruited to the damaged heart through the CCL2-CCR2 axis^9^.

The results of this study have revealed an anti-fibrotic effect of OSM (Supplementary Fig. 21). We still need to be careful in using OSM or an OSMR agonist for the management of cardiac fibrosis, since it may have a potential to drive intestinal inflammation^43^. Therefore, selective delivery of OSM to the heart such as coronary artery injection seems to be necessary to manage cardiac fibrosis effectively. It should also be noted that the fibroblast activation processes in TAC model may be quite different from that in myocardial infarction. Further investigation, therefore, will help to understand the context dependent activation processes of cardiac fibrosis in more detail.

## Methods

### Mice and surgical procedures

All animal experiments were approved by the University of Tokyo Ethics Committee for Animal Experiments or King’s College London Ethical Review Process Committee and UK Home Office (Project License No. PPL70/7260). The procedures are strictly adhered to the guidelines for animal experiments of the University of Tokyo or the Guidance on the Operation of the Animals (Scientific Procedures) Act, 1986 (UK Home Office). C57BL/6 mice were purchased from Crea Japan (Tokyo, Japan) or Envigo (Blackthorn, UK). B6.129-Hif1a^tm3Rsjo/J^ (HIF-1α^flox/fox^) mice, B6;129-Osmr^tm1.1Nat/J^ (OSMR^flox/flox^) mice, B6.129P2-Lyz2^tm1(cre)Ifo/J^ (LysM-cre) mice, B6.Cg-Tg(Tek-cre)12Flv/J (Tie2-cre) mice and B6.Cg-Tg(Col1a1-cre/ERT2)1Crm/J (Col1a1-creERT) mice were purchased from The Jackson Laboratory (Bar Harbor, Maine, USA). HIF-1α^flox/fox^, LysM-Cre and Tie2-cre mice were backcrossed to C57BL/6 background. Mice were housed in a specific pathogen-free facility with a 12-h light/12-h dark cycle. Tamoxifen (1 mg) (Sigma–Aldrich, St-Louis, USA) was injected intraperitoneally for 7 days to induce cre-mediated recombination in Col1a1-creERT mice. Corn oil was administered in control groups. Seven days following cessation of tamoxifen animals were subjected to TAC procedure. Male mice 7–12 weeks of age were used for TAC operation. TAC operation was performed as follows, a 27-gauge needle was placed next to the aortic arch, the suture was tied firmly two times around the 27-gauge needle and the aorta, and the needle was removed^34^. Sham operated mice underwent the same procedure but without constriction. Mice were closely observed and euthanized quickly, at a humane end point (no locomotion or body weight loss exceeding 20% of the initial body weight).

### Cells, reagents, recombinant proteins, and antibodies

Mesenchymal stem cell line C3H10T1/2 cells were purchased from American Type Culture Collection. C3H10T1/2 cells were grown in DMEM (Dulbecco’s Modified Eagle Medium) medium containing 10% fetal bovine serum (HyClone, GE Healthcare Japan, Tokyo, Japan) in a 37 °C incubator (5% CO_2_). TEPMs were collected from the peritoneal cavity 4 days after the intraperitoneal injection of 3% thioglycollate solution (Fluka, Sigma–Aldrich, St Louis, MO, USA) using female mice. TEPMs were grown in RPMI1640 (GIBCO, Life technologies, Carlsbad, CA, USA) medium containing 10% fetal bovine serum (HyClone) in a 37 °C incubator (5% CO_2_). Adult male mice at the age of 6–8 weeks were used for the isolation and cultivation of cardiac fibroblasts^44^. Two or three hearts of ventricles were isolated and quickly minced into small pieces. After incubation at 37 °C for 30 min with DMEM containing Liberase TH (25 μg per ml, Sigma–Aldrich) and elastase (1.2 U per ml, Worthington Biochemical Corporation, Freehold, NJ, USA), the cell suspensions were pelleted and washed with PBS(-). All cell suspensions were plated on a collagen-coated dish (Cosmo Bio, Tokyo, Japan) in DMEM supplemented with 10% of fetal bovine serum. After overnight incubation, nonadherent cells were removed, and adherent cells were cultivated. Primary fibroblasts at passage 1 or 2 were used for experiments.

Recombinant mouse OSM (catalog no. 495-MO-025), mouse IL6 (catalog no. 406-ML-005), and human TGF-β1 (catalog no. 240-B-010) were all purchased from R&D Systems (Minneapolis, MN, USA). Recombinant mouse CTGF (catalog no. RPA010MU01) was purchased from Cloud-Clone Corp. (Katy, TX, USA). Recombinant mouse CXCL2 (catalog no. 582502), mouse LIF (catalog no 554002) and mouse CXCL3 (catalog no. 590802) were purchased from Bio Legend (San Diego, CA, USA). All recombinant proteins used in cell culture were diluted in 0.5% BSA solution (Sigma, catalog no. A8806-1G).

APC-CD11b (M1/70) (diluted 1:1000, 17-0112-82; eBioscience, PE-F4/80 (BM8) (diluted 1:1000, 123110; BioLegend, FITC-Ly6C (HK1.4) (diluted 1:1000, 128006; BioLegend, Pacific Blue-Ly6G (1A8) (diluted 1:1000, 127612; BioLegend) were used for the FACS analysis. Mouse OSM antibody (AF-495-NA; R&D) or normal Goat IgG control (AB-108-C; R&D) was used as a neutralizing antibody or control IgG in vivo. Human OSM antibody (diluted 1:500, NBP1-87768; R&D) and mouse OSM antibody (diluted 1:500, bs-5095R; BIS) were used for the OSM staining in human and mouse heart tissues respectively. Mouse CD11b antibody (diluted 1:2000, ab133357; Abcam, Cambridge, UK) was used for the CD11b staining in mouse heart tissues.

Exposure of cell cultures to hypoxia (1% O_2_) was performed in the hypoxic workstation In Vivo_2_ 500 (Ruskinn Technology, Bridgend, UK). The serum free culture medium of TEPMs was changed 12 h after hypoxic exposure. Follow which, TEPMs were exposed to hypoxia for 12 h, and their supernatant was collected and used in the analysis of fibroblast activation.

### Preparation of clodronate liposomes

Clodronate liposomes were generated as follows^45^. After 8 mg cholesterol was dissolved in 10 ml chloroform, 0.86 ml of phosphatidylcholine solution (containing 86 mg phosphatidylcholine) was added. The chloroform phase was removed by low vacuum rotation (1.257 × *g*) evaporation. The phospholipid film was dispersed in 10 ml of aqueous solution containing 0.6 M clodronate by gentle rotation at room temperature. The milky white suspension was kept at room temperature for 2 h under nitrogen gas. The suspension was sonicated in a waterbath sonicator for 3 min and kept overnight at 4 °C. After centrifugation (10,000 × *g* for 15 min), the solution under the white band of liposomes was removed using a Pasteur pipette. The clodronate liposomes are washed three times using sterilized PBS (Centrifugation at 25,000 × *g* for 30 min). Finally, the pellet was resuspended in 4 ml sterilized PBS.

### Flow cytometry

All flow cytometric analyses and sorting were performed using FACS aria II (BD) and FlowJo software (Tree Star). To isolate cells from hearts, whole heart was cut into small pieces in DMEM (containing 10% fetal bovine serum). The collected tissue was incubated for 2 h with collagenase type I (1 mg per ml, Worthington Biochemical Corporation) and elastase (0.74 U per ml, Worthington Biochemical Corporation) in Hanks’ buffered saline solution (HBSS). The suspension was passed through a 22-gauge needle and washed in ice-cold PBS(−) containing 5% fetal bovine serum. After removing any erythrocytes using BD PharmLyse (BD), the samples were subjected to flow cytometric analysis.

The heart samples were obtained 45 min. after intraperitoneal injection of pimonidazole (Pimo, 60 mg per kg) (hpi, Burlington, MA, USA). Intracellular incorporation of Pimo was analyzed by multicolor FACS with a fluorescein (FITC)-conjugated anti-Pimo antibody (diluted 1:1000, hpi)

### Intravital visualization of hypoxic signals in heart

Hypoxic state is visualized using two photon microscope and phosphorescence probe, LOX-1 (Scivax Life Sciences, Tokyo, Japan)^46^. Anesthesized and mechanically-ventilated mice were administrated with Hoechst 33342 (10 mg per kg, Invitrogen (Thermofisher), blue), fluorescein labeled Griffonia Simplicifolia Lectin I (GSL I) isolectin B4 (green, Vector laboratories, FL-1201, 50 μg for each mouse), and LOX-1 hypoxic probes (100 μg for each mouse, red). Imaging of living heart was performed by two photon microscope (Nikon-A1RMP, Nikon), femtosecond infrared lasers (Chameleon Vision-II, Coherent), and ×40 water-immersion objective lens (NA1.15, Nikon). Signal intensity for LOX-1 was automatically quantified and statistically analyzed by NIS-elements software (Nikon).

### RNA isolation and quantitative RT-PCR

For gene expression analysis, total RNA was purified from cultured cells using RNeasy kits (Qiagen, Tokyo, Japan) according to the manufacturer’s instructions. Complementary DNA was synthesized using the SuperScript III First-Strand Synthesis System (Life Technologies). Quantitative real-time PCR (RT-PCR) analyses were conducted using the LightCycler system (Roche Diagnostic, Tokyo, Japan), with 18S rRNA serving as the internal control. Primer sequences of the analyzed mouse genes are shown in Supplementary Table 1.

### Western blotting

Cell extracts were separated on TGX FastCast acrylamide Kit, 7.5% (BIO RAD Laboratories Inc.), and then transferred to PVDF membrane (Millipore, Bedford, MA, USA). After blocking in 5% dry milk in Tris-buffered saline with Tween 20 (0.1%) (TBS-T), the membrane was incubated with primary antibodies (1:1000) followed by incubation with horseradish peroxidase-conjugated secondary antibodies (1:2000). Detection was performed using ECL Prime (GE Healthcare, Logan, UT, USA) according to the manufacturer’s instructions. The following antibodies were used: SMAD2 (#5339, Cell Signaling Technology, Danvers, Ma, USA), Phospho-SMAD2 (Ser245/250/255) (#3104, Cell Signaling Technology, Danvers, MA, USA), Phospho-SMAD2 (Ser465/467) (#3108, Cell Signaling Technology, Danvers, MA, USA), Lamin A/C (#2032, Cell Signaling Technology, Danvers, MA, USA), ERK (#4695, Cell Signaling Technology, Danvers, MA, USA), Phospho-ERK (#4370, Cell Signaling Technology, Danvers, MA, USA), goat anti-rabbit IgG^−^HRP (#7074, Cell Signaling Technology, Danvers, MA, USA). After the exposure to phospho ERK1/2 or phosphor SMAD2 C-terminal antibody, the same membrane was exposed with anti-ERK or Lamin A/C antibody. Whole images of western blot images are shown in Supplementary Figs. 22–24.

### ChIP assay

TEPMs were cross-linked for 10 min by 1% paraformaldehyde. After neutralization using 0.2 M glycine solution, TEPMs were collected and suspended in SDS lysis buffer with cOmplete protease inhibitor cocktail (1873580, Roche). Samples were subjected to fragmentation using Sonifier (Branson, Dansbury, USA). Sonicated samples were immunoprecipitated with the HIF-1α antibody (NB100-134, Novus Biologicals). ChIP samples were quantified with RT-PCR using specific primer pairs (Supplementary Table 1).

### RNA sequencing (RNA-seq) analysis

TEPMs were isolated from HIF-1α KO or cre negative littermate controls. We exposed TEPMs to hypoxia (1% O_2_) or normoxia and collected total RNA at 4 h and 12 h time points, then performed transcriptome analysis (RNA-seq.). Single-end RNA-seq libraries were prepared using a TruSeq RNA Sample Prep Kit (Illumina, San Diego, CA). Sequencing runs were performed on an Illumina Genome Analyzer IIx (Illumina, San Diego, CA, USA). The reads were aligned to the mm9 mouse genome using TopHat (ver. 2.0.0). Generation of gene expression data, normalization and gene annotation processes were performed using Genomatix Genome Analyzer (Genomatix, Munich, Germany). The normalized expression value (NE-value) was calculated as follow: the number of reads per a gene × 10^7^ per the number of mapped reads in the genome × gene length.

### Identification of hypoxia-inducible secretory factors

To identify hypoxia-inducible secretory factors, we performed procedures as follows. Initially, we selected the genes with NE-value more than 0.01 in at any time points (Analytic genes: 11,000 genes). Second, we picked up the genes those maximum NE-vale were upregulated more than 2-folds in hypoxia (Hypoxia upregulated genes: 866 genes). Third, we selected the genes in which hypoxic induction of their expression is abrogated in HIF-1α KO TEPMs (HIF-1α dependent genes: 614 genes)). Finally, genes that have gene ontology (GO) terms of ‘cytokine activity’, ‘chemokine activity’ or ‘growth factor activity’ were extracted (http://www.geneontology.org/) (Hypoxia-inducible secretory factors: 11 genes).

### Histological studies of mouse and human heart tissues

Written informed consent was obtained from all participants. The study protocol was approved by the Ethics Committee of The Cardiovascular Institute (Tokyo, Japan), and strictly adhered to the ethical regulations. Heart tissues were obtained from heart failure patients, when they underwent heart surgery or endomyocardial biopsy. The patients with prior medical history of myocardial infarction were excluded. Immunohistochemistry were performed in each specimen. Masson trichrome staining and Sirius Red/ Fast Green staining were performed to calculate perivascular and interstitial fibrotic area.

### Statistical analysis

All data are shown as average with SD. Comparison between two groups was analyzed using unpaired two-tailed *t*-test with Welch’s correction. Differences among more than two groups were analyzed using one-way or two-way ANOVA followed by Dunnett’s multiple comparisons test or Sidak’s multiple comparison test or Kruskal–Wallis test. *P* -values of less than 0.05 were considered to be statistically significant. Kaplan–Meyer analysis, followed by a long-rank test, was used to analyze the survival of mice. Linear regression analysis was used for the correlation between percent of fibrotic area and the number of OSM-positive cells in the heart tissues. All statistical analyses were performed using Prism 6 for Windows (version 6.07, GraphPad, San Diego, CA, USA).

## Supplementary information

Supplementary Information

## Data Availability

All data contained in the manuscript are available from the corresponding author upon reasonable request. The RNA-seq data of peritoneal macrophages have been deposited in DDBJ BioProject under accession number DRA008230.
