# Supplementary material for: Macrophage hypoxia signaling regulates cardiac fibrosis via Oncostatin M
Source: Nat Commun. 2019 Jun 27;10:2824. doi: 10.1038/s41467-019-10859-w (PMC6597788; doi:10.1038/s41467-019-10859-w)
Supplement: Supplementary file 1 — Supplementary Information [file 41467_2019_10859_MOESM1_ESM.pdf]

Macrophage hypoxia signaling regulates cardiac fibrosis via Oncostatin M

Abe. et al 2019

Supplementary figures and table.

# Supplementary Figure-1

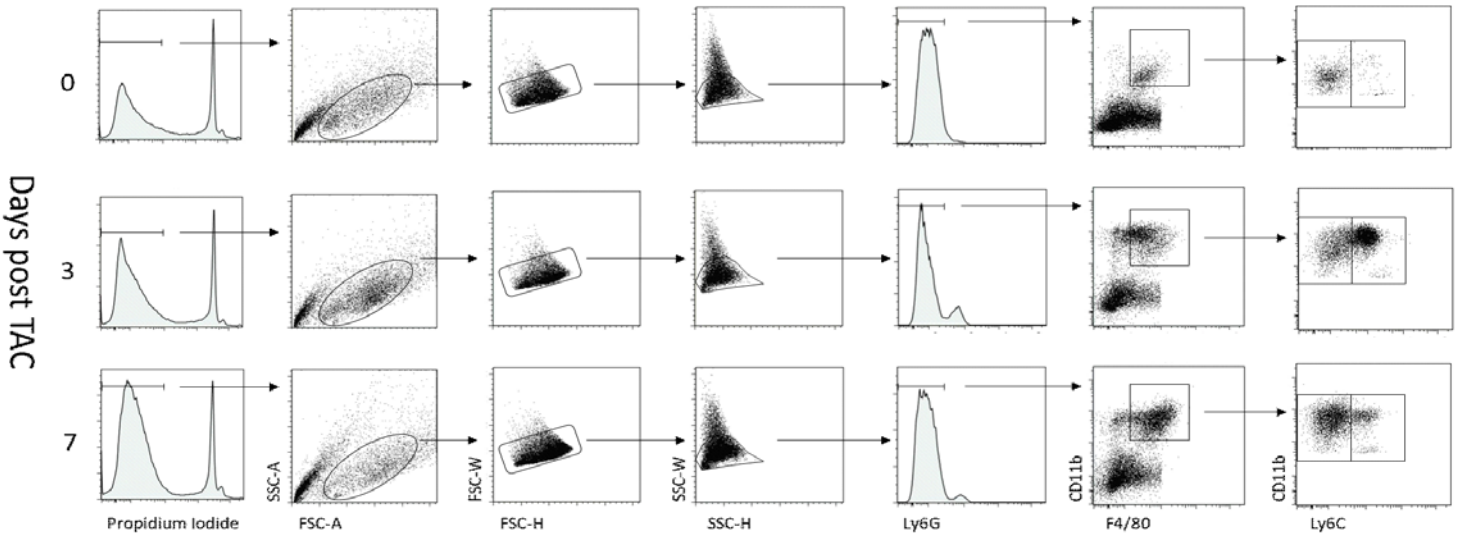


Representative gating strategy to analyze cardiac infiltration of Mϕ (CD11b$^+$, F4/80$^+$, Ly6G$^-$ mononuclear cells) was presented. Cardiac Mϕ can be devided into two populations, depending on the expression level of Ly6C (Ly6C$^{hi}$ and Ly6C$^{lo}$).

# Supplementary Figure-2

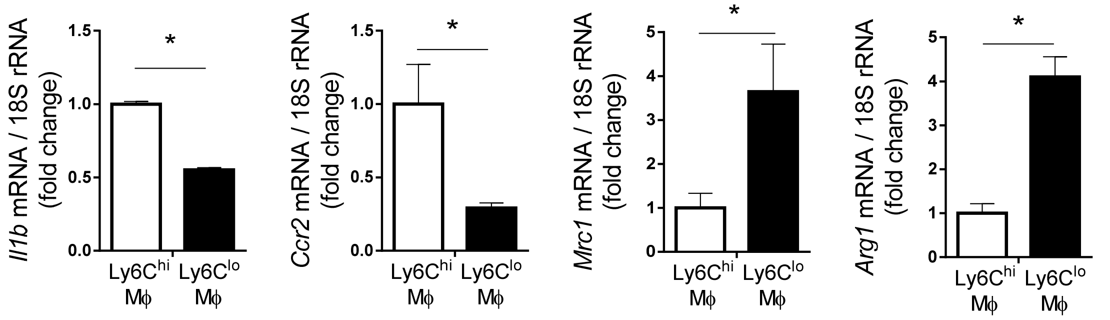


The expression levels of Mϕ activation marker genes were assessed by quantitative PCR. Data show the mean and the SD of technical triplicates from a representative experiment. Two-tailed t-test with Welch's correction was used for the statistical analysis (*Il1b* (t = 32.41, df = 3.737), *Ccr2* (t = 4.513, df = 2.068), *Mrc1* (t = 4.039, df = 2.382), *Arg1* (t = 10.63, df = 2.876)). *, $p < 0.05$.

# Supplementary Figure-3

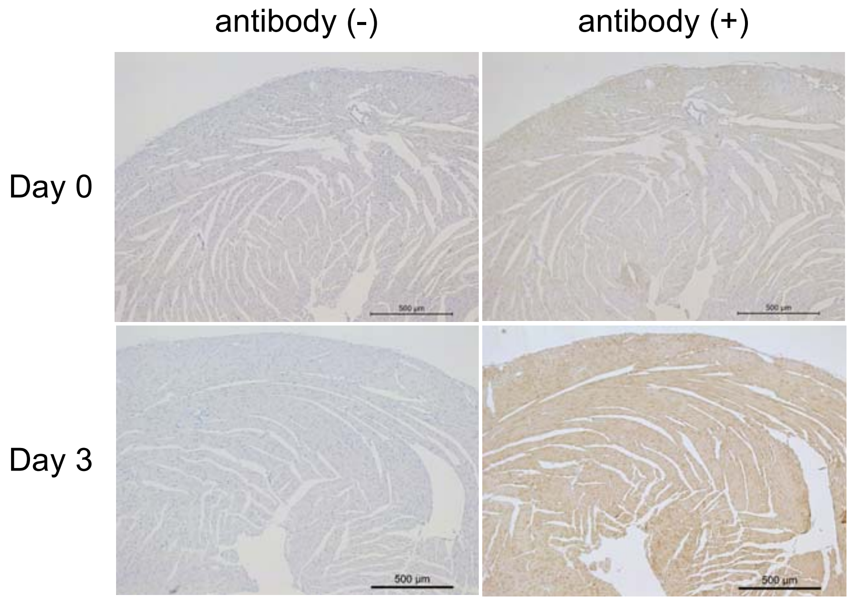


Immunohistological stainings of pimonidazole were performed using cardiac tisseues from TAC operated mice (day 0, day 3). The heart samples were obtained 45 min. after intraperitoneal injection of pimonidazole (Pimo, 60 mg per kg). Pimo antibody (diluted 1:100, hpi, Burlington, MA, USA) was used for the Pimo staining in mouse heart tissues. Scale bar = 500 μm.

# Supplementary Figure-4

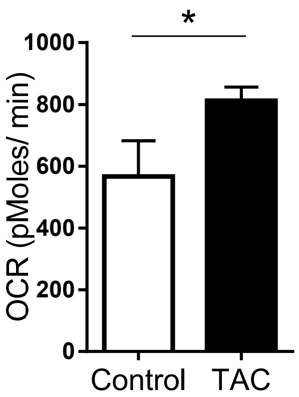


The oxygen consumption rate (OCR) of cardiomyocytes was measured in Seahorse XF24 Extracellular Flux Analyzer (Agilent Technologies, USA). Cardiomyocytes were isolated by the Langendorff perfusion method from 6-8 weeks male mice 3 days after TAC or sham operation. Following euthanization, hearts were immediately excised and placed in ice-cold, Ca-free "Basal Solution" (130 mM NaCl, 5.4 mM KCl, 0.5 mM MgCl2, 0.33 mM NaH2PO4, 25 mM HEPES and 22 mM D-glucose, pH adjusted to 7.4). The aorta was cannulated with and tied firmly to a blunt-ended 23-gauge needle attached to the Langendorff apparatus. The perfusion protocol began with Basal Solution containing 0.4 mM EGTA, perfused for 1 min at 4 ml per min. The perfusate was then switched to "Solution 1" (1 mg per ml type II collagenase (Worthington, USA), 0.05 mg per ml protease from Streptomyces griseus (Sigma, USA) and 0.1 mM Ca added to the Basal Solution), and perfused for 9 min at 3 ml per min. After completing the 10-minutes perfusion, the left ventricle was separated from the great vessels, atria and right ventricle. The collected left ventricle was carefully triturated in "Solution 2" (Basal Solution supplemented with 1 mg per ml type II collagenase, 0.05 mg per mL protease, 0.3 mM Ca and 2 mg per ml bovine serum albumin (Sigma, USA)), achieving highly viable, rod-shaped cardiomyocytes. Gentle centrifugation at 20 g for 3 min and pellet resuspension was repeated twice, using buffer solutions with incrementing Ca concentrations, 0.5 mM ("Solution 3") and 1.0 mM ("Solution 4"), in order to achieve stepwise restoration of physiological Ca levels. Prior to seeding, the isolated cardiomyocytes were again gently pelleted and resuspended to the XF assay medium (unbuffered DMEM supplemented with 25 mM D-glucose). Following cell counting, the cardiomyocyte suspension was titrated to $1 \times 10^4$ perl cell density, 675 μL of which was seeded onto laminin-coated Seahorse V7 tissue culture plates. Cardiomyocytes were allowed to attach for 2 hours in a $CO_2$-free incubator maintained at 37 degree. For both TAC and sham conditions, the basal oxygen consumption rate was measured in Seahorse XF24 Analyzer. Data show the mean and the SD of technical triplicates from a representative experiment. The measurement was repeated at least three independent experiments.Two-tailed t-test with Welch's correction was used for the statistical analysis (t = 3.873, df = 4.044). *, $p < 0.05$.

# Supplementary Figure-5

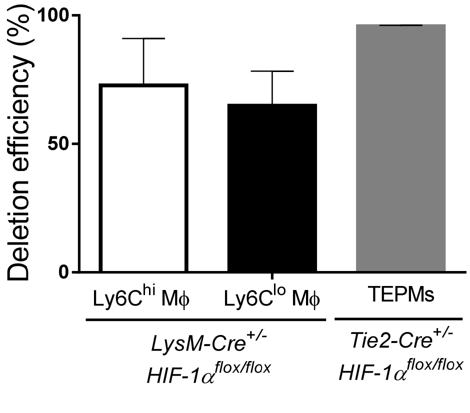


Deletion efficiency of *HIF-1α* mRNA in Mϕ was examined. Total RNA was isolated from sorted Mϕ (*LysM-Cre*$^{+/-}$ *HIF-1α*$^{flox/flox}$) and TEPMs (*Tie2-Cre*$^{+/-}$ *HIF-1α*$^{flox/flox}$), and subjected to quantitative PCR with primers spanning the deleted region as well as primers for an undeleted control gene for normalization. Efficiency of deletion was calculated by quantitative PCR. Data show the mean and the SD of technical triplicates from a representative experiment.

# Supplementary Figure-6

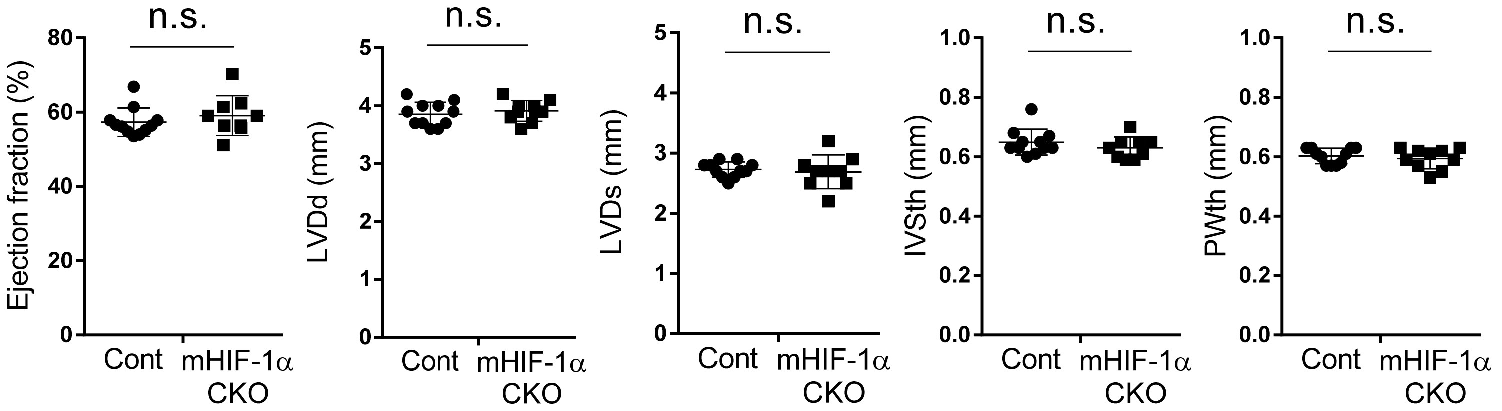


Data represent the transthoracic echocardiography data in unoperated control (cont) (n = 11) and mHIF-1α CKO (n = 9). Ejection fraction, left ventricular end-diastolic dimension (LVDd), left ventricular dimeter at end systole (LVDs), thickness of interventricular septum (IVSth) and thickness of posterior LV wall (PWth) are measured. The Mann-Whitney U test was used to compare differences between cont and mHIF-1α CKO mice. Data show the mean and the standard deviation (error bar). n.s., not statistically significant.

# Supplementary Figure-7

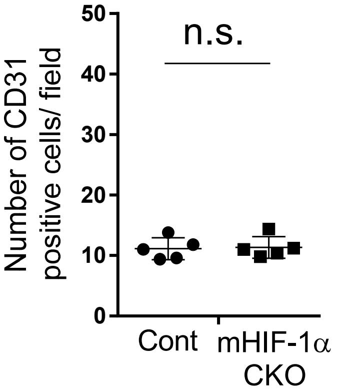


The average number of  CD31 positive cells within 5 fields were counted 14 days after TAC operation. Control (cont) (n = 5) and mHIF-1α CKO (n = 5). Mouse CD31 antibody (DIA-310; diluted 1:200, Dianova, Hamburg, Germany) was used for the CD31 stainig in mouse heart tissues. The Mann-Whitney U test was used to compare differences between cont and mHIF-1α CKO. Data show the mean and the standard deviation (error bar). n.s., not statistically significant.

# Supplementary Figure-8

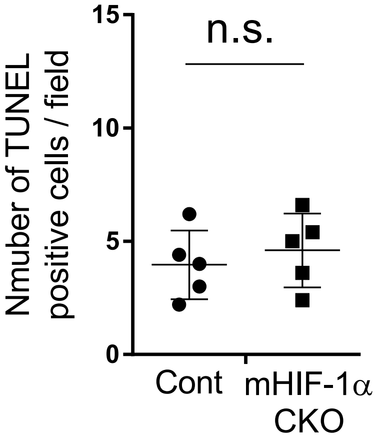


The average number of  TUNEL positive cells within 5 fields were counted 3 days after TAC operation. Control (cont) (n = 5) and mHIF-1α CKO (n = 5). Cardio TACS in situ apoptosis detection kit (R & D systems) was used for the TUNEL staining. The Mann-Whitney U test was used to compare differences between cont and mHIF-1α CKO. Data show the mean and the standard deviation (error bar). n.s., not statistically significant.

# Supplementary Figure-9

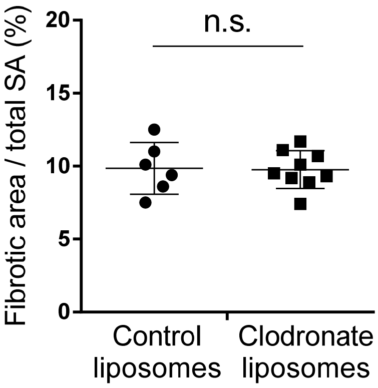


Clodoronate liposomes (200 μl) and control liposomes (200 μl) were injected intravenously 2 days after TAC operation. Masson's trichrome staining was performed using cardiac tissues of mHIF-1α CKO mice 14 days after TAC operation. Fibrotic area was calculated compared to the total surface are (SA). The Mann-Whitney U test was used to compare differences between control liposomes (n = 6) and clodronate liposomes (n = 9) groups. Data show the mean and the standard deviation (error bar). n.s., not statistically significant.

# Supplementary Figure-10

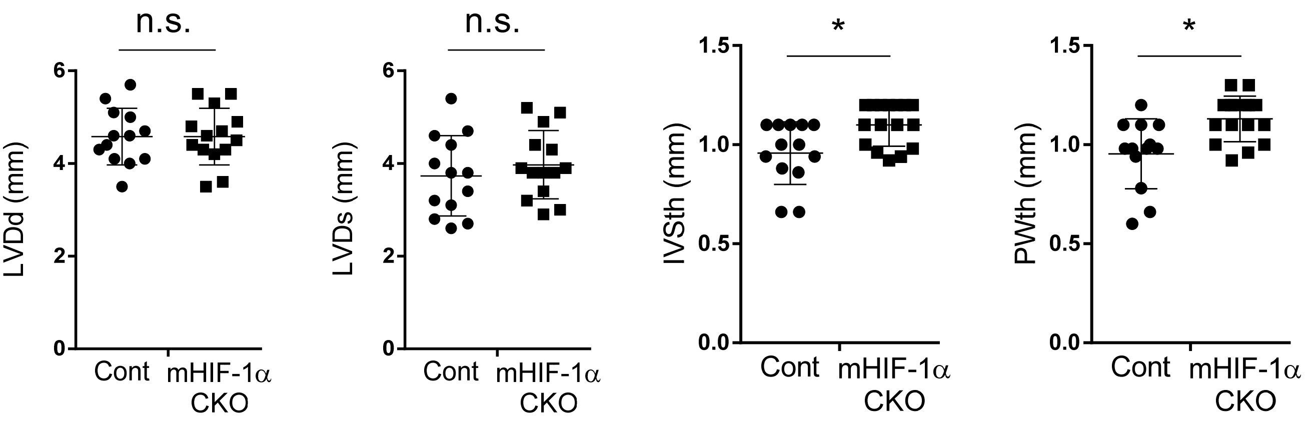


Data represent the transthoracic echocardiography data 28 days after TAC operation in control (cont) (n = 13) and mHIF-1α CKO mice (n = 16). Left ventricular end-diastolic dimension (LVDd), left ventricular dimeter at end systole (LVDs), thickness of interventricular septum (IVSth) and thickness of posterior LV wall (PWth) are measured. The Mann-Whitney U test was used to compare differences between cont and mHIF-1α CKO mice. Data show the mean and the standard deviation (error bar). n.s., not statistically significant. *, $p < 0.05$.

# Supplementary Figure-11

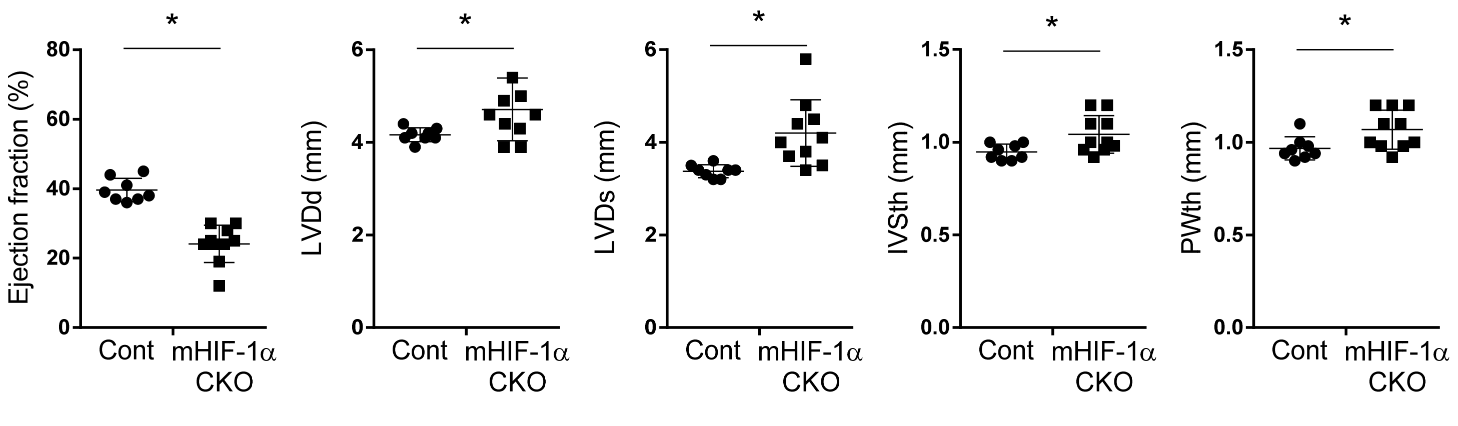


Data represent the transthoracic echocardiography data 42 days after TAC operation in control (cont) (n = 8) and mHIF-1α mice (n = 10). Ejection fraction, left ventricular end-diastolic dimension (LVDd), left ventricular dimeter at end systole (LVDs), thickness of interventricular septum (IVSth) and thickness of posterior LV wall (PWth) are measured. The Mann-Whitney U test was used to compare differences between cont and mHIF-1α CKO mice. Data show the mean and the standard deviation (error bar). *, $p < 0.05$.

# Supplementary Figure-12

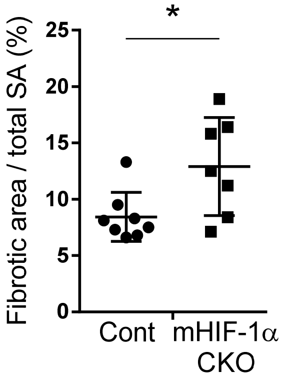


Myocardial infarction model was performed as follows. Mice were intubated and ventilated. After exposing the heart at the fourth left intercostal space, the left coronary artery was permanently ligated with an 8-0 nylon.Fibrotic area was calculated compared to the total surface area (SA) using cardiac tissues 14 days after myocardial infarction operated mice. The Mann-Whitney U test was used to compare differences between cont (n = 8) and mHIF-1α CKO (n = 7). Data show the mean and the standard deviation (error bar). *, $p < 0.05$.

# Supplementary Figure-13

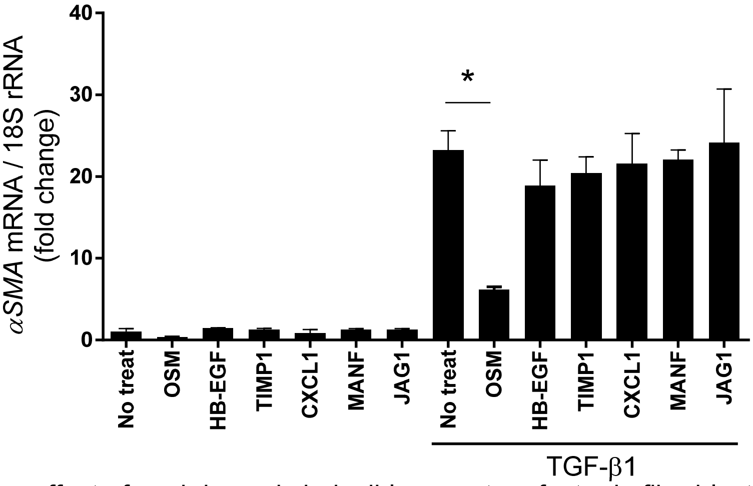


The effect of each hypoxic inducible secretory factor in fibroblasts activation was examined (20 ng per ml for HB-EGF, TIMP1, CXCL1, MANF, JAG1 and 10 ng per ml for OSM). Thirty minutes after pretreatment with hypoxia inducible secretory factors, C3H/10T1/2 cells were stimulated with TGF-β1 (2.5 ng per ml, 12 h) and the relative expression of αSMA mRNA was calculated. Recombinant mouse OSM (catalog no. 495-MO-025) and human TGF- β1 (catalog no. 240-B-010) were all purchased from R&D Systems (Minneapolis, MN, USA). Recombinant mouse HB-EGF (catalog no. 268-10191-1) was purchased from RayBiotech (Katy, TX, USA). Recombinant mouse TIMP1 (catalog no. 593702) was purchased from Bio Legend (San Diego, CA, USA). Recombinant mouse CXCL1 (catalog no. 200-23) was purchased from Shenandoah Biotechnology (Warwick, PA, USA). Mouse recombinant MANF (catalog no. CYT-827) was purchased from Prospec-Tany Technogene (Rehovot, Israel). JAG1 (catalog no. ab109346) was purchased from abcam (Cambridge, UK). All recombinant proteins used in cell culture were diluted in 0.5% BSA solution (Sigma, catalog no. A8806-1G). The one-way ANOVA and Dunnett's multiple comparisons test were used for the statistical analysis (F (6, 14) = 10.17). Data show the mean and the SD of technical triplicates from a representative experiment. *, $p < 0.05$.

# Supplementary Figure-14

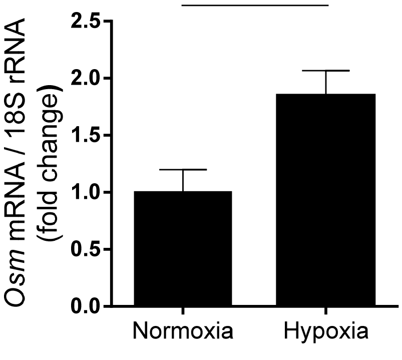


The transcript levels of *Osm* in bone marrow-derived macrophages (BMDMs) were assessed by quantitative PCR. Bone marrow cells were collected from tibia of adult male mice at the age of 6-8 weeks. Monocyte-colony stimulating factor (M-CSF) was added at day 0 (40 ng per ml), day 3 (40 ng per ml) and day 6 (20 ng per ml). M-CSF was purchased from R & D Systems (Minneapolis, MN, USA). We exposed BMDMs to hypoxia (1% O2) or normoxia and collected total RNA at 12 h time point. Data show the mean and the SD of technical triplicates from a representative experiment. Two-tailed t-test with Welch's correction was used for the statistical analysis (t = 4.256, df = 2.131). *, $p < 0.05$.

# Supplementary Figure-15

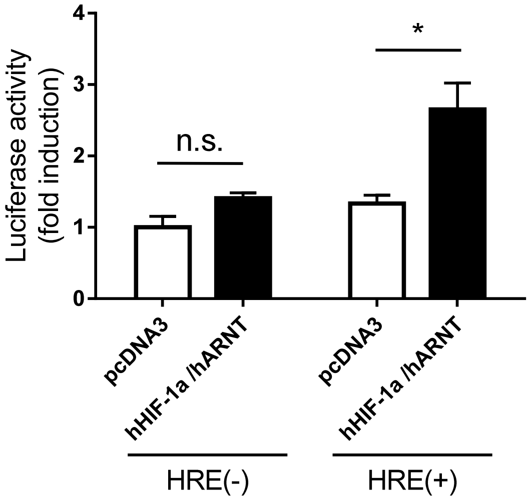


Co-transfection of HIF-1α and aryl hydrocarbon receptor nuclear translocator (ARNT) significantly activated the promoter activity of pGL3mOSM(-1023 to +129)/HRE. Data show the mean and the SD of technical triplicates from a representative experiment. The two-way ANOVA and Sidak's multiple comparison test was performed for the statistical analysis ($F (1, 8) = 49.37$). n.s., not statistically significant. *, $p < 0.05$ vs pcDNA3.

Construction of reporter plasmids. phHIF-1 and phARNT were generated as described[1]. Murine liver genomic DNA was isolated using Wizard Genomic DNA Purification Kit (Promega) and used for construction of Osm reporter plasmids. A fragment of the promoter region of Osm (-1023 to +129bp relative to TSS (transcription start site)) was amplified using a pair of primers (forward;5'-GATCGCAGATCTCGAACTGGGTCCTGGTACTCTGGC-3', reverse;5'-CTAGCCCGGGCTCGATGCTGTGGCTTCCAAGCATGGC-3'). PCR product was subcloned into the Xho1 site of the promoter in pGL3-Basic vector (Promega) using In-Fusion HD Cloning Kit (Z9633N, TaKaRa Clontech, Japan), yielding pGL3mOSM(-1023 to +129). A fragment containing the HRE region of Osm (-4151 to -3772 bp relative to TSS) was amplified using a pair of primers (forward;5'- GGATCCAGCCTCAGCAGAGCCAGTCCGA -3', reverse;5'-GGATCCGGTTGGCTATGGGGACAGGAGTG -3') and inserted at BamH1 site of the enhancer in pGL3mOSM(-1023 to +129), yielding pGL3mOSM(-1023 to +129)/HRE.

The luciferase assays were performed by transfecting reporter constructs into COS7 cells using Fugene HD (Promega, Madison, WI, USA) according to manufacturer's instrument. The CMV-beta-galactosidase plasmid was co-transfected as an internal control for transfection efficiency. The cells were harvested using passive lysis buffer (Promega) at 48 h after transfection and assayed for the luciferase activity by means of the Dual-Glo Luciferase assay system (Promega, Madison, WI, USA) and a luminometer (TriStar2 Multimode Reader LB 942, Berthold Technologies GmbH & Co. KG, Bad Wildbad, Germany).

# Supplementary Figure-16

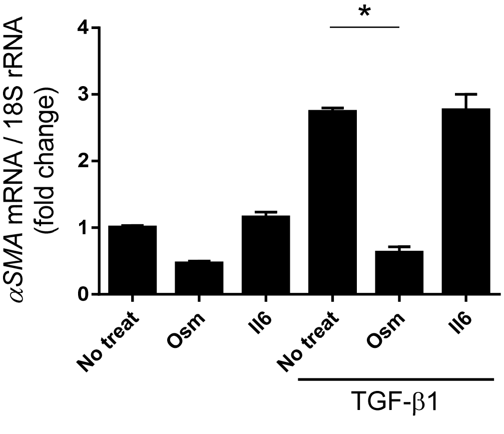


After pretreatment with the OSM (10 ng per ml) or IL6 (20 ng per ml), primary cariac fibroblasts were stimulated with TGF-β1 (2.5 ng per ml, 12 h) and the relative expression level of αSMA mRNA was calculated. Data show the mean and the SD of technical triplicates from a representative experiment. The one-way ANOVA and Dunnett's multiple comparisons test was used for the statistical analysis ($F (2, 6) = 197.2$). *, $p < 0.05$.

# Supplementary Figure-17

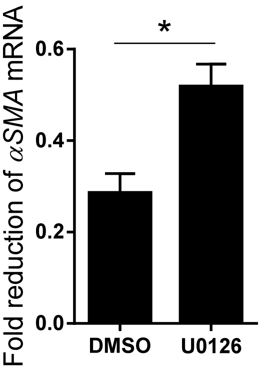


After pretreatment with OSM (10 ng per ml, 30 min) in the presence of U0126 (20 μM, 60 min) or DMSO, primary cardiac fibroblasts were stimulated with TGF-β1 (2.5 ng per ml, 12 h) and the relative expression level of αSMA mRNA was calculated. Data show the mean and the SD of technical triplicates from a representative experiment. Two-tailed t-test with Welch's correction was used for the statistical analysis (t = 6.142, df = 3.924). *, $p < 0.05$.

# Supplementary Figure-18

A.

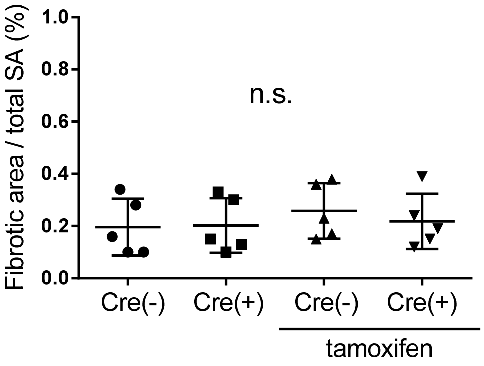


B.

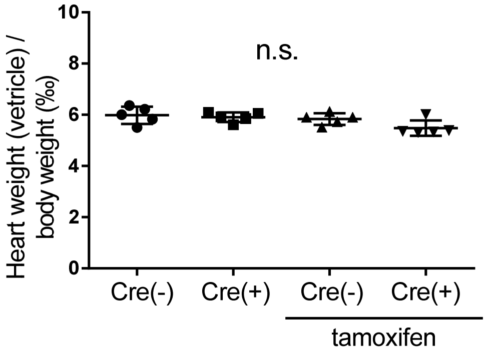


(A) Fibrotic area in unoperated mice was calculated compared to the total surface area (SA) using cardiac tissues of fOSMR CKO mice. (B) Data represent the heart weight (ventricles) / body weight (‰) using unoperated caridac tissues of fOSMR CKO mice. The Kruskal-Wallis test was used for the statistical analysis. (cre(-), tamoxifen(-) : n = 5, cre(+), tamoxifen(-) : n = 5, cre(-), tamoxifen(+) : n = 5, cre(+), tamoxifen(+) : n = 5 ), Data show the mean and the standard deviation (error bar). n.s., not statistically significant.

# Supplementary Figure-19

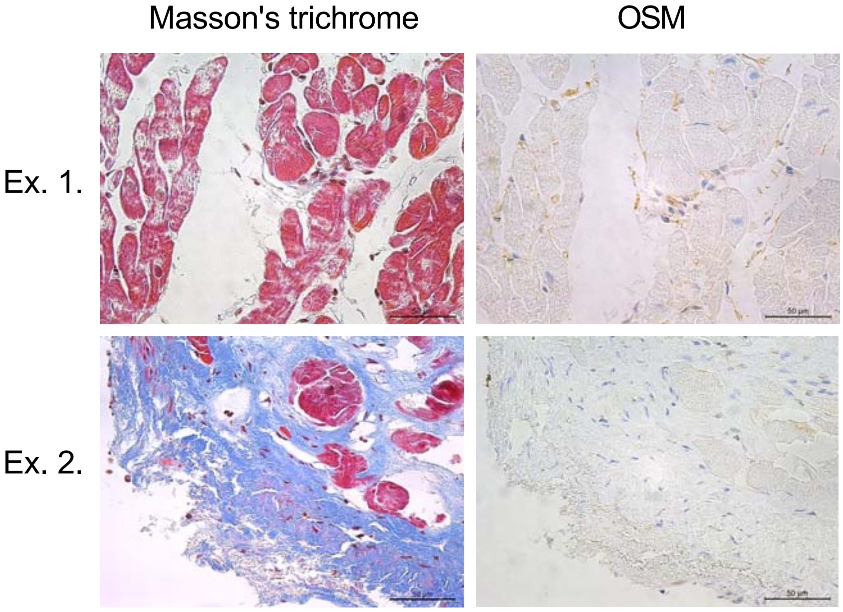


Masson's trichrome staining and immunohistological staining of OSM were perfomed using heart specimens from human heart failure patients (n = 18). Staining for representative individuals are shown. (OSM staining: OSM brown, nucleus blue) (area of cardiac fibrosis, Ex. 1. 12%, Ex. 2. 56%), (number of OSM positive cells per view, Ex. 1. 20, Ex. 2. 8). Scale bar = 20 μm.

# Supplementary Figure-20

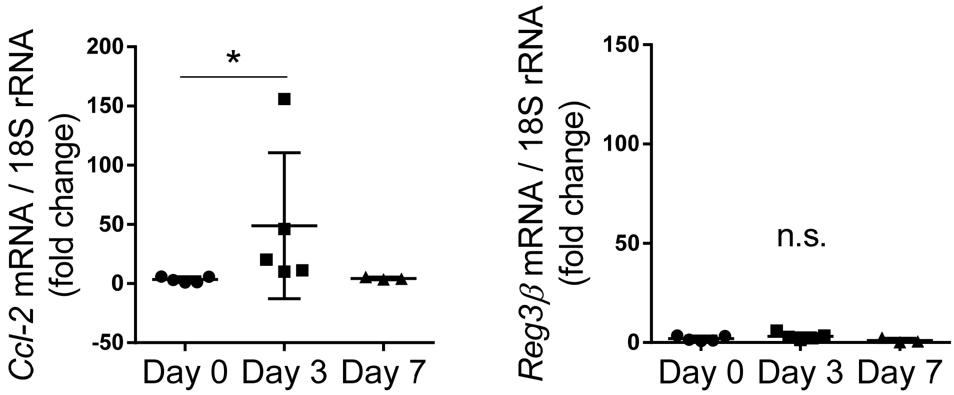


RT-qPCR analysis of *Ccl-2* and *Reg3β* mRNA expression level in the heart tissues after TAC operation. Data show the mean and the standard deviation (error bar). The Kruskal-Wallis test was used for the statistical analysis (Day 0 : n = 5, Day 3 : n = 5, Day 7 : n = 3). n.s., not statistically significant. *, $p < 0.05$.

# Supplementary Figure-21

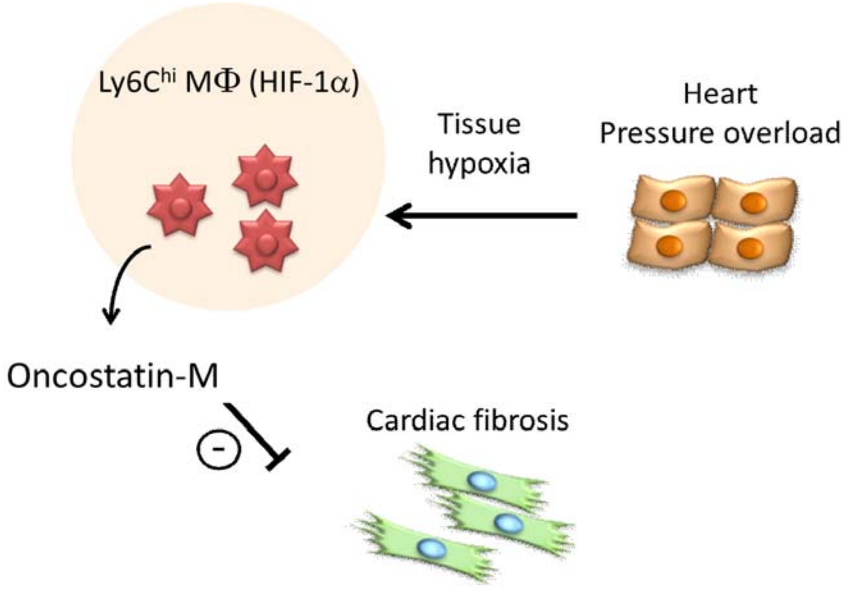

# Supplementary Figure-22

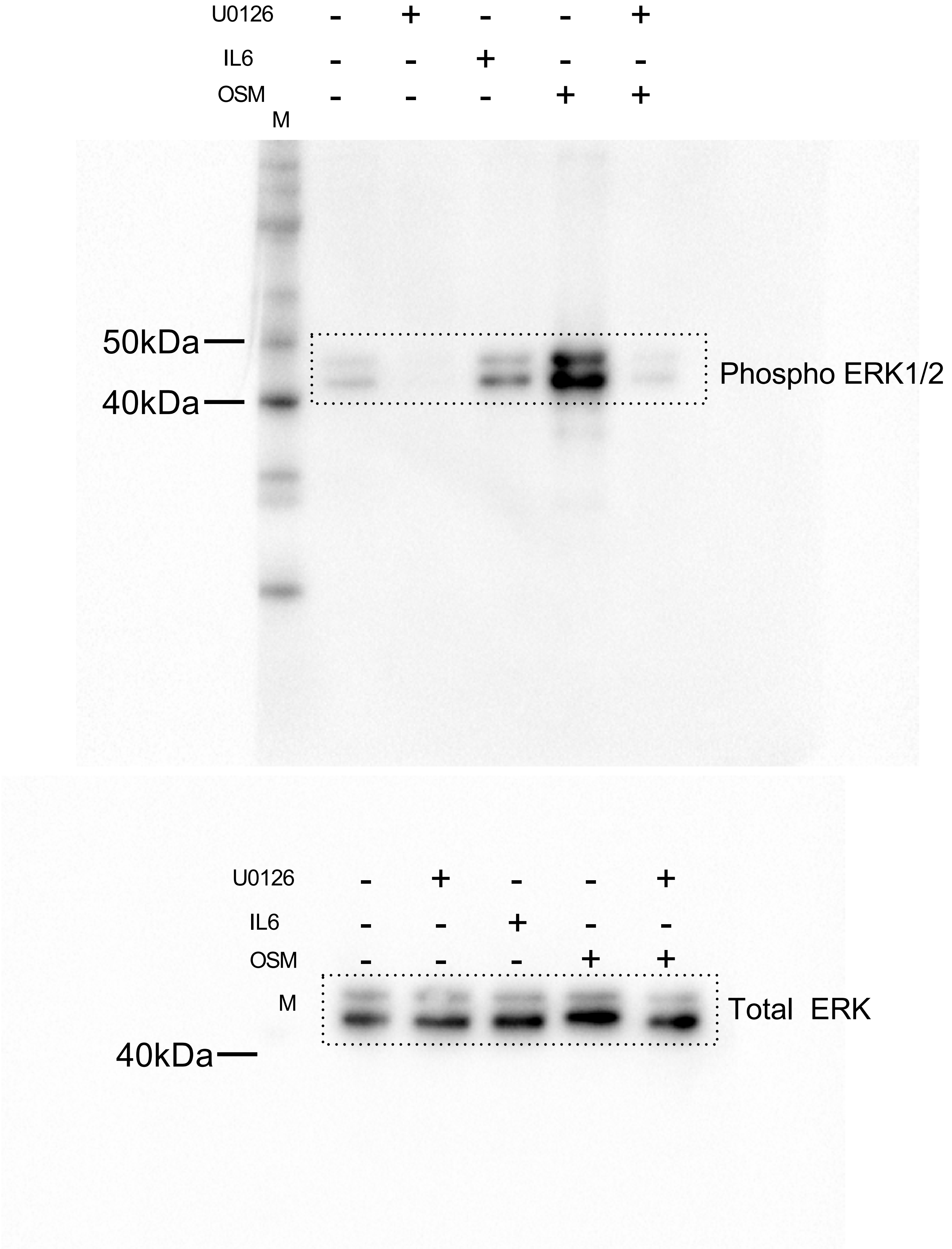


Whole images of western blots.

# Supplementary Figure-23

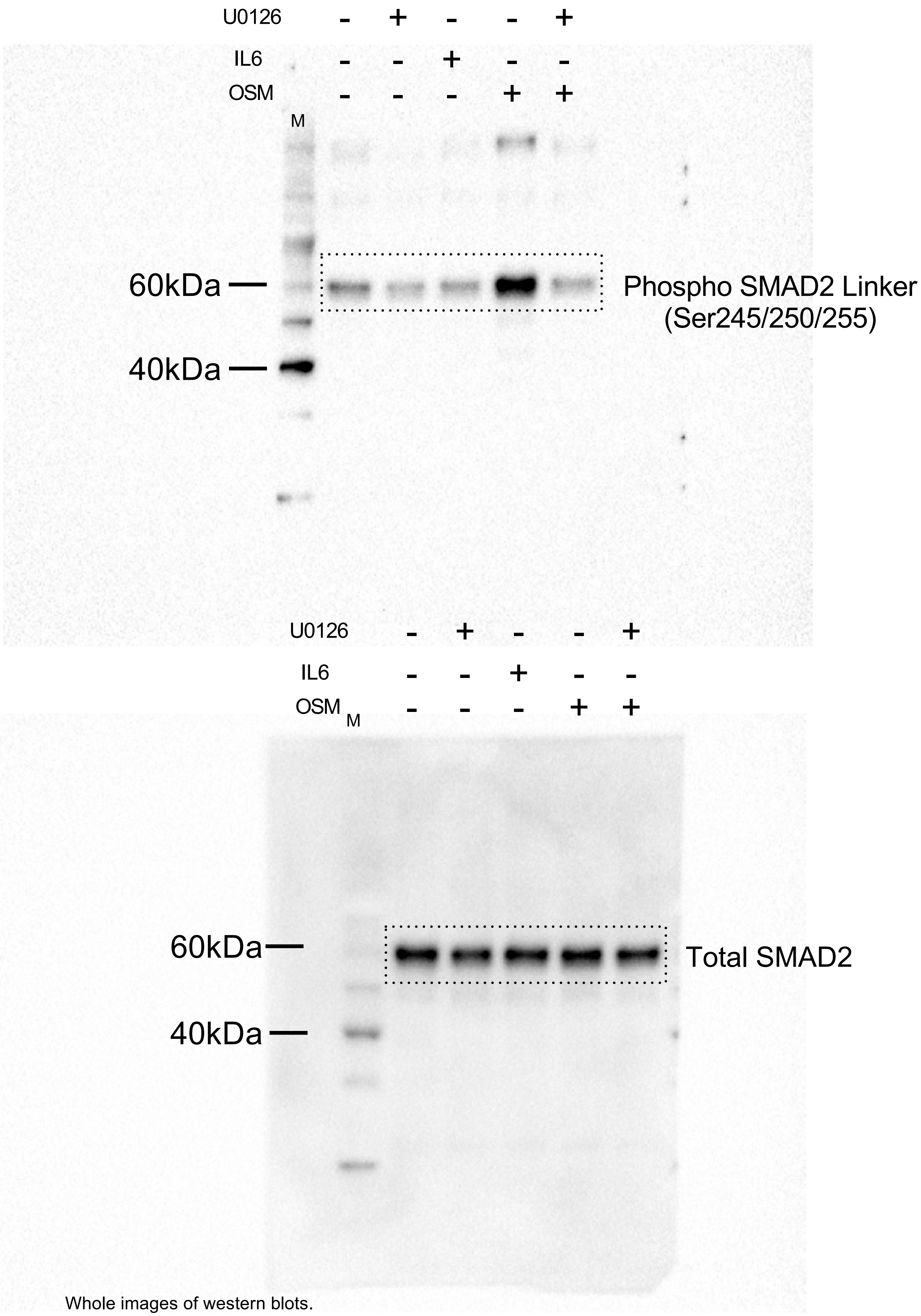


Whole images of western blots.

# Supplementary Figure-24

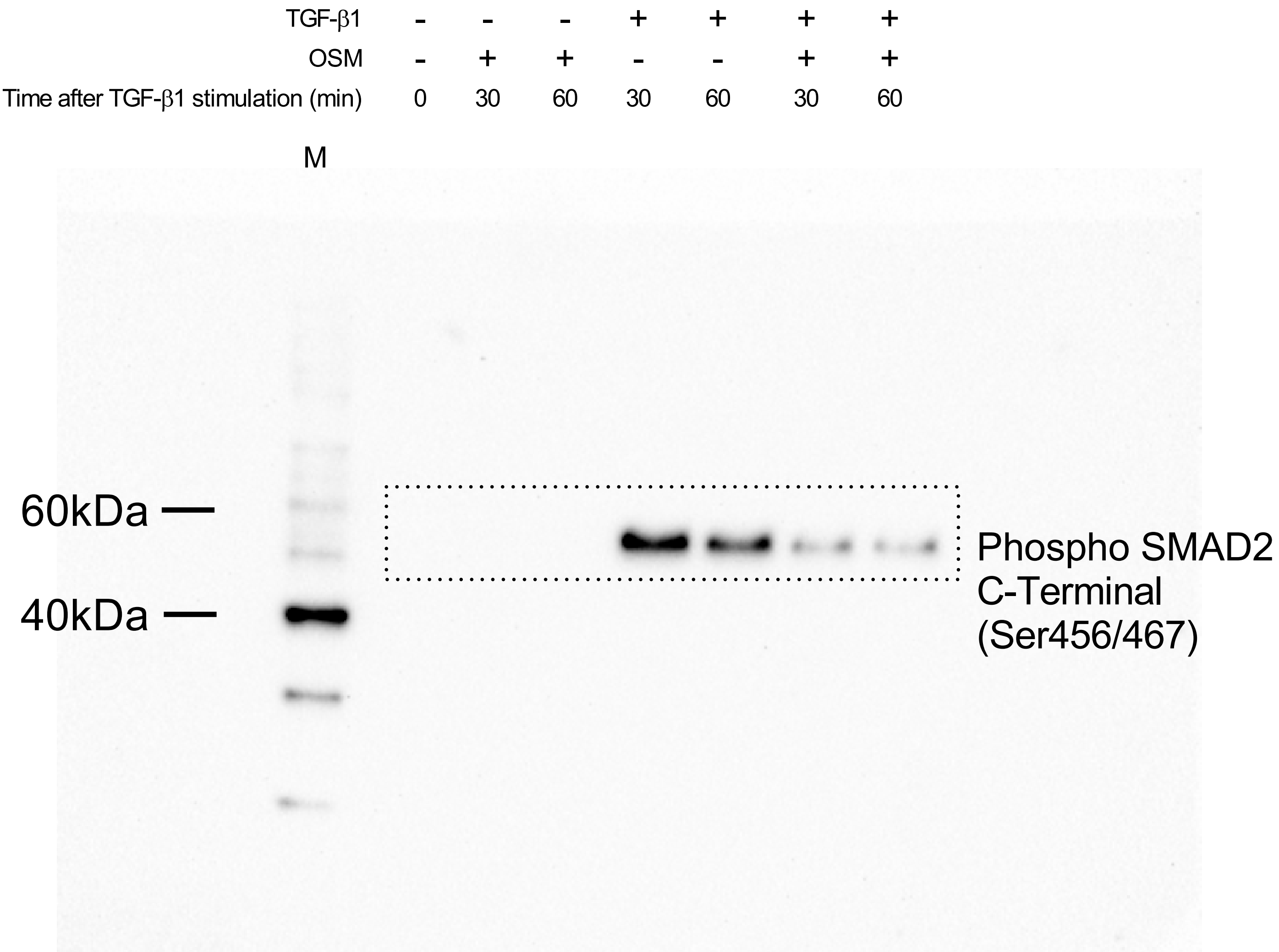


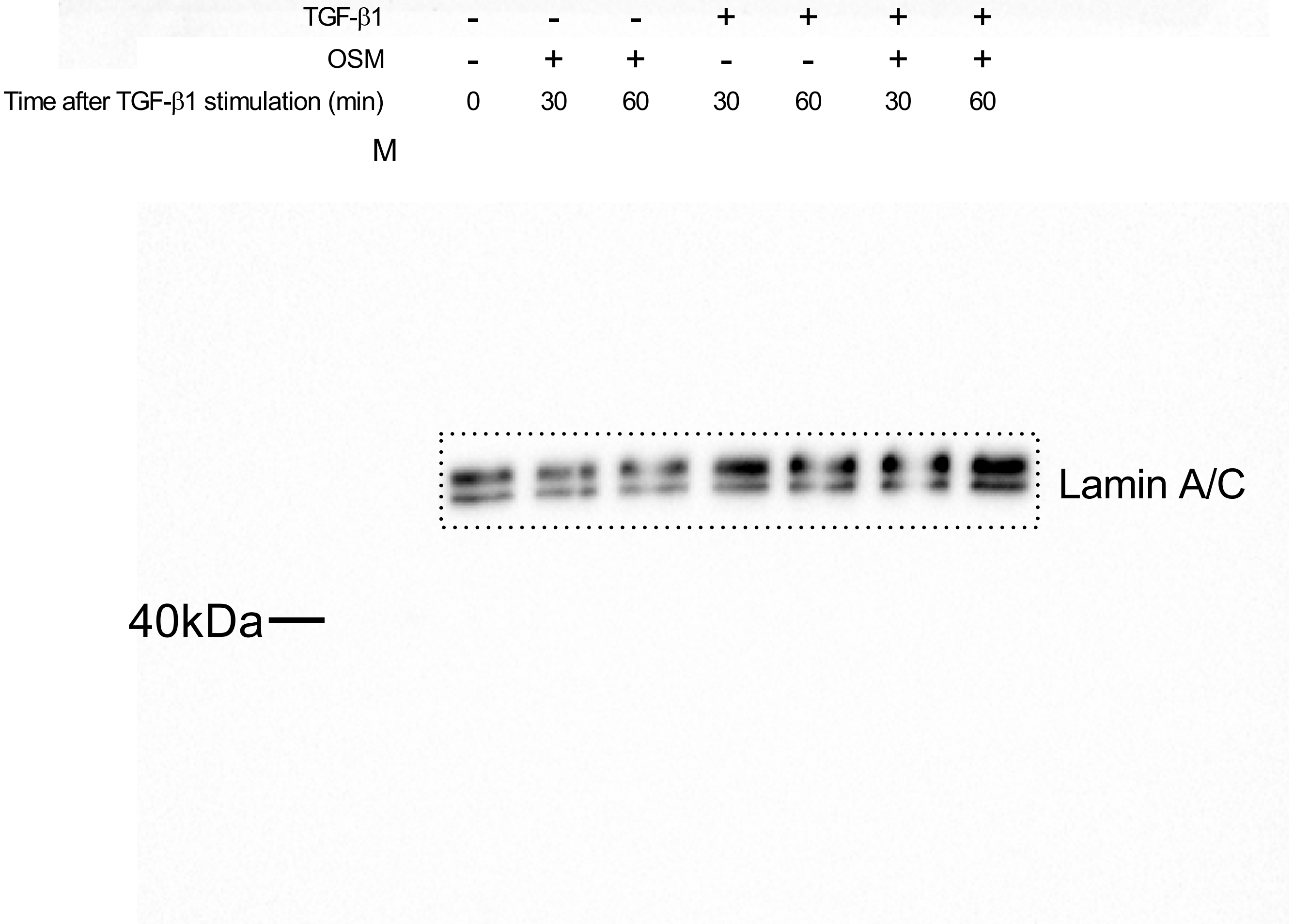


Whole images of western blots.

# Supplementary Table-1

| Primer Sequences for RNA analysis | | | Primer Sequences for ChIP PCR analysis | |
|---|---|---|---|---|
| *18S* -F | CGAAAGCATTTGCCAAGAAT | | (Primer 1 ) *Pgk1* distal region | |
| *18S* -R | AGTCGGCATCGTTTATGGTC | | F | GGCATTAGGGCATTCAGTTC |
| *Il1b* -F | TGAGCACCTTCTTTTCCTTCA | | R | TCCACTCTGAATCCTGGTGA |
| *Il1b* -R | TTGTCTAATGGGAACGTCACAC | | (Primer 2) | |
| *Mrc1* -F | CACTCATCCATTACAACCAAAGC | | *Pgk1* F | CACCTTCTACTCCTCCCCTAGTCA |
| *Mrc1* -R | CAGGAGGACCACGGTGAC | | *Pgk1* R | CACGAGACTAGTGAGACGTGCTACTT |
| *Acta2* -F | GCATCCACGAAACCACCTA | | (Primer 3) | |
| *Acta2* -R | CACGAGTAACAAATCAAAGC | | *Osm* F1 | TGACTGTTGCAAGCCTTTCC |
| *Hif-1α* -F | GATTCGCCATGGAGGGC | | *Osm* R1 | TCACTACCATCCAGGCAAAGC |
| *Hif-1α* -R | AGACTCTTTGCTTCGCCGAG | | (Primer 4) | |
| *Hif-1α* -F | GATTCGCCATGGAGGGC (for checking the deletion efficiency) | | *Osm* F2 | GCAGTTCTGAGGAAGCAAACC |
| *Hif-1α* -R | AGACTCTTTGCTTCGCCGAG (for checking the deletion efficiency) | | *Osm* R2 | AGGAAAGGCTTGCAACAGTC |
| *Ccr2*- F | ACCTGTAAATGCCATGCAAGT | | | |
| *Ccr2* -R | TGTCTTCCATTTCCTTTGATTTG | | | |
| *Arg1* -F | AACACGGCAGTGGCTTTAACC | | | |
| *Arg1* -R | GGTTTTCATGTGGCGCATTC | | | |
| *Ccl2* -F | GTTGGCTCAGCCAGATGCA | | | |
| *Ccl2* -R | AGCCTACTCATTGGGATCATCTTG | | | |
| *Reg3β* -F | GAGGCCTGGAGGACACCTCGT | | | |
| *Reg3β* -R | TTGTCCCTTGTCCATGATGCTCTTC | | | |
| *Osm* -F | GGCAACTGAGCAAGCCTCAC | | | |
| *Osm* -R | CTGAGCCCATGAAGCGATGG | | | |

**Supplementary Reference**

1. Maemura K, et al. Generation of a dominant-negative mutant of endothelial PAS domain protein 1 by deletion of a potent C-terminal transactivation domain. J Biol Chem 274, 31565-31570 (1999).
